# An Old Crystallization Technique as a Fast, Facile, and Adaptable Method for Obtaining Single Crystals of Unstable “Li_2_TCNQF_4_” and New Compounds of TCNQ or TCNQF_4_: Syntheses, Crystal Structures, and Magnetic Properties

**DOI:** 10.1021/acs.cgd.3c00160

**Published:** 2023-05-26

**Authors:** Slavomíra Šterbinská, Mariia Holub, Erik Čižmár, Juraj Černák, Lawrence Rocco Falvello, Milagros Tomás

**Affiliations:** † Faculty of Sciences, Institute of Chemistry, Department of Inorganic Chemistry, P. J. Šafárik University in Košice, Moyzesova 11, 041 54 Košice, Slovakia; ‡ Faculty of Sciences, Institute of Physics, P. J. Šafárik University in Košice, Park Angelinum 9, 041 54 Košice, Slovakia; § Instituto de Nanociencia y Materiales de Aragón (INMA) and Departamento de Química Inorgánica, CSIC-Universidad de Zaragoza, Zaragoza 50009, Spain; ∥ Instituto de Síntesis Quimica y Catálisis Homogénea (ISQCH), Departamento de Química Inorgánica, Pedro Cerbuna 12, University of Zaragoza−CSIC, E-50009 Zaragoza, Spain

## Abstract

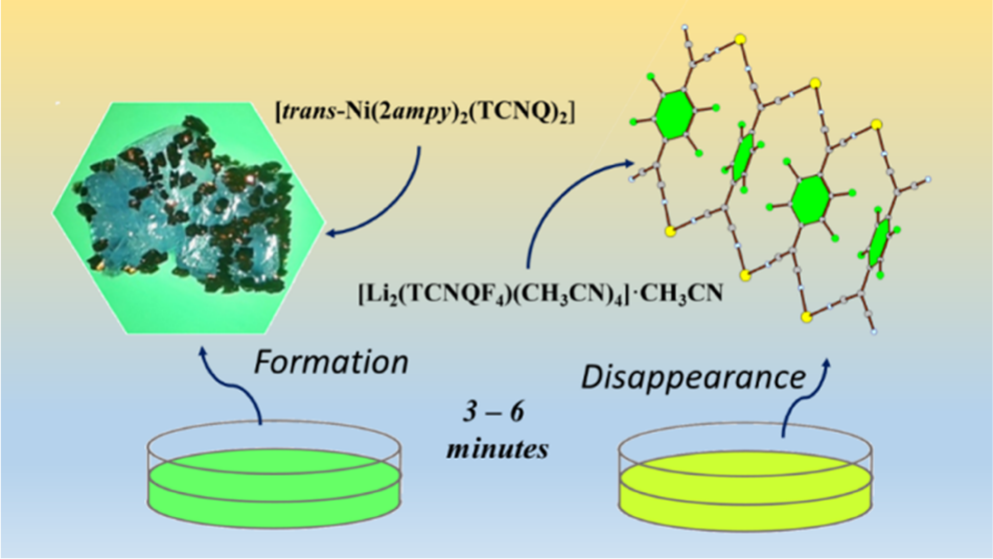

Detailed structural information is essential for understanding the properties of TCNQ and TCNQF_4_ compounds (TCNQ = 7,7,8,8-tetracyanoquinodimethane; TCNQF_4_ = 2,3,5,6-tetrafluoro-7,7,8,8-tetracyanoquinodimethane). The ineludible requirement of obtaining crystals of a size and quality sufficient to yield a successful X-ray diffraction analysis has been challenging to satisfy because of the instability of many of these compounds in solution. Crystals of two new complexes of TCNQ, [*trans*-M(2*ampy*)_2_(TCNQ)_2_] [M = Ni (**1**), Zn (**2**); 2ampy = 2-aminomethylpyridine], as well as unstable [Li_2_(TCNQF_4_)(CH_3_CN)_4_]·CH_3_CN (**3**), can be prepared in minutes by a horizontal diffusion technique and can be harvested easily for X-ray structural studies. Compound **3**, previously described as “Li_2_TCNQF_4_,” forms a one-dimensional (1D) ribbon. Compounds **1** and **2** can also be obtained as microcrystalline solids from methanolic solutions of MCl_2_/LiTCNQ/2*ampy*. Their variable-temperature magnetic studies confirmed a contribution of strongly antiferromagnetically coupled pairs of TCNQ^•—^ anion radicals at higher temperatures with exchange coupling *J*/*k*_B_ = −1206 K and *J*/*k*_B_ = −1369 K for **1** and **2**, respectively, estimated using a spin dimer model. The presence of magnetically active anisotropic Ni(II) atoms with *S* = 1 in **1** was confirmed, and the magnetic behavior of **1**, representing an infinite chain of alternating *S* = 1 sites and *S* = 1/2 dimers, was described by a spin-ring model suggesting ferromagnetic exchange coupling between Ni(II) sites and anion radicals.

## Abstract 2

A horizontal diffusion technique facilitates the crystallization of three compounds with the TCNQ radical anion or diamagnetic TCNQF_4_. The magnetism of the heterospin Ni(II) complex and that of the isostructural Zn(II) analogue are compared. The same procedure permits the capture of the ephemeral lithium cation-containing polymer [Li_2_TCNQF_4_(CH_3_CN)_4_]_*n*_·*n*CH_3_CN.

## Introduction

Compounds based on TCNQ [TCNQ = 7,7,8,8-tetracyanoquinodimethane; IUPAC 2,2′-(cyclohexa-2,5-diene-1,4-diylidene)dipropanedinitrile] and its derivatives manifest an ample variety of physical properties, with their electronic and magnetic behavior having been the subject of innumerable studies. Key to understanding the properties of TCNQ derivatives, particularly in solids, is the determination of their structures, with accurate information on bonding and intermolecular interactions involving TCNQ^*n*–^ (*n* = 0, 1, 2, and intermediate between 0 and 2), which in turn requires the preparation of crystals adequate for structure determination, usually by in-house X-ray diffraction.

Some of the widely used procedures for the preparation of crystals such as solvent evaporation, solvent exchange, and vertical diffusion of different solutions require the compound to be in solution for hours to days, or even months because a slow evaporation or diffusion process favors the formation of fewer nuclei with more crystal growth.^1^

**Scheme 1:**
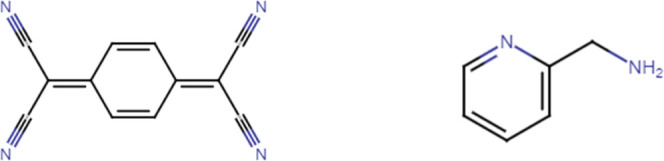
Chemical Structures of TCNQ and 2*ampy*

TCNQ (Scheme 1) as a diamagnetic solid can be reduced easily, yielding the TCNQ^•–^ anion radical (AR), and by further reduction, a diamagnetic dianion TCNQ^2–^ can be formed.^2^ TCNQ in the form of the AR is interesting from a magnetic point of view since the presence of an unpaired electron makes it magnetically active (*S* = 1/2). The magnetic properties of numerous compounds based on the TCNQ AR have been studied in detail.^3−12^ Besides magnetic properties, TCNQ-containing compounds were studied as promising semiconducting materials,^13,14^ materials for quantum computing,^15^ electric capacitors,^16^ and gas sensors,^17^ and as porous materials for gas storage and separation.^18^

The TCNQ AR can act as a simple counter anion, or due to the presence of four cyanide groups, it can act as a ligand in coordination complexes; in the latter case, the TCNQ AR can be bound as a terminal ligand *via* an N atom from one of the cyanide groups, or it can act as a μ_2_-, μ_3_-, or μ_4_-bridging ligand.^2^

From a magnetic point of view, complexes containing magnetically active 3d or 4f central atoms and TCNQ AR are heterospin systems in which the overall magnetic properties are determined by the magnetic properties of both constituents and by their interaction. In addition, the magnetic properties will also be affected by the arrangement of the TCNQ ARs; in the case of the frequently occurring π-dimerization of TCNQ units, their strong antiferromagnetic (AFM) coupling leads to a singlet ground state of the π-dimerized unit.^19^ Heterospin complexes with Ni(II) possess the combination of spins *S* = 1 and *S* = 1/2, which can be considered as analogues to Ni(II)/Cu(II) heterobimetallic complexes (*S* = 1, *S* = 1/2).^20^

The Cambridge Structural Database^21^ records more than 30 complexes of Ni(II) with TCNQ. Among these complexes, monodentate coordination of TCNQ AR to the Ni(II) central atom is infrequent, having been observed to date only in the complexes [Ni*L*^R^(TCNQ)_2_] (*L*^R^ = 1,8-(C_2_H_4_OH)_2_-1,3,6,8,10,13-hexaazacyclotetradecane),^22^ [Ni(*cyclam*)(TCNQ)_2_] (*cyclam* = 1,4,8,11-tetraazacyclotetradecane),^23^ and [Ni(*L*2)(TCNQ)_2_](TCNQ)·CH_3_COCH_3_ (*L*2 = pentaazamacrocyclic ligand containing the 1-hexadecyl alkyl chain).^24^ In all of these complexes, the TCNQ ARs occupy relative *trans*-positions in the octahedral coordination sphere of the central Ni(II) atom, while a *cis*-arrangement of TCNQ ARs was observed only in [Ni(*trien*)(TCNQ)_2_] (*trien* = 1,4,7,10-tetraazadecane),^23^ as forced by the character of the blocking ligand *trien*.

It was observed some time ago^25^ that recrystallization of these compounds frequently produces their degradation, and thus for structure analysis, it is important to obtain the compounds directly as single crystals to avoid the necessity of recrystallization. For instance, Li^+^ salts of TCNQ or TCNQF_4_ (TCNQF_4_ = 2,3,5,6-tetrafluoro-7,7,8,8-tetracyanoquinodimethane) can be used as reactants for the preparation of TCNQ/TCNQF_4_ compounds; however, there have been few reports of structures of Li^+^ with TCNQ or TCNQF_4_. Besides some organic salts in which Li^+^ is bonded to crown ethers,^26−28^ there are just two structures; in one of them, TCNQ is bonded to Zn,^29^ and in the other one,^30^ each Li^+^ cation is coordinated by four TCNQF_4_^2–^ groups forming a two-dimensional (2D) metal–organic framework.

Within our broader interest in the crystal structures and magnetic properties of heterospin Ni(II) complexes with the TCNQ AR,^12,31^ we have prepared a novel heterospin complex [Ni(2*ampy*)_2_(TCNQ)_2_] (**1**; 2*ampy* = 2-aminomethyl-pyridine) with two TCNQ ARs coordinated to the Ni(II) central atom. This compound has been prepared in two different ways, as microcrystals from a methanol solution of NiCl_2_·6H_2_O and 2*ampy* with LiTCNQ and as single crystals of sufficient quality for X-ray structure determination from the reaction in methanol of crystals of an insoluble 2*ampy* coordination compound, [Ni(2*ampy*)_2_(NO_3_)_2_], with soluble LiTCNQ, in a fast, facile procedure designed to avoid decomposition. The phase identity of the compounds obtained through the two different procedures was established using X-ray powder diffraction. We have further tested this procedure with the preparation and structure analysis of single crystals of [Li_2_(TCNQF_4_)(CH_3_CN)_4_]·CH_3_CN (**3**). The preparation of this compound in acetonitrile and its instability were described some ten years ago,^32^ at which time it was reported that attempts to grow crystals suitable for diffraction analysis were unsuccessful.

We also report the crystal structure at three temperatures (100, 170, and 293 K) of the new Ni(II) compound (**1**), along with its magnetic properties. For the sake of comparison, especially with the aim of elucidating the contribution of the TCNQ AR to the magnetic properties, we have also synthesized and structurally and magnetically characterized the isostructural Zn(II) complex [Zn(2*ampy*)_2_(TCNQ)_2_] (**2**).

## Experimental Section

### Materials

NiCl_2_·6H_2_O, ZnCl_2_, TCNQ, TCNQF_4_, LiI, methanol, 2*ampy*, and FomblinY oil (d: 1.92 g/mol at 25 °C, average M.W. 6500) were purchased from commercial sources and used as received. LiTCNQ, [Ni(2*ampy*)_2_(NO_3_)_2_], and [Zn(2*ampy*)_2_(NO_3_)_2_] were prepared according to published procedures.^25,33,34^

### Synthesis and Crystallization of [*trans*-Ni(2*ampy*)_2_(TCNQ)_2_]

To a methanolic solution of NiCl_2_·6H_2_O (5 mL, 0.3 mmol; 0.079 g) were added consecutively with vigorous stirring, a methanolic solution (3 mL) of 2*ampy* (0.6 mmol; 0.07 mL) and a methanolic solution (10 mL) of LiTCNQ (0.72 mmol; 0.168 g). After 10 min, the resulting solid was separated by filtration and washed with methanol and diethyl ether. Yield: 71%.

CHN analysis (exp./calc. in %) for C_36_H_24_N_12_Ni (*M* = 683.38 g/mol): C, 62.50/63.27; H, 3.80/3.54; N, 24.31/24.60.

FT-IR (ATR, cm^–1^): 3315m, 3260m, 3028w, 2178vs, 2159m, 1606w, 1572vs, 1501vs, 1433m, 1337vs, 1286w, 1253w, 1216w, 1185vs, 1123s, 1103m, 1021s, 984s, 954w, 929w, 878w, 825vs, 755vs, 718vs, 643s, 543m, 511w, 482vs, 449w.

**Figure 1:**
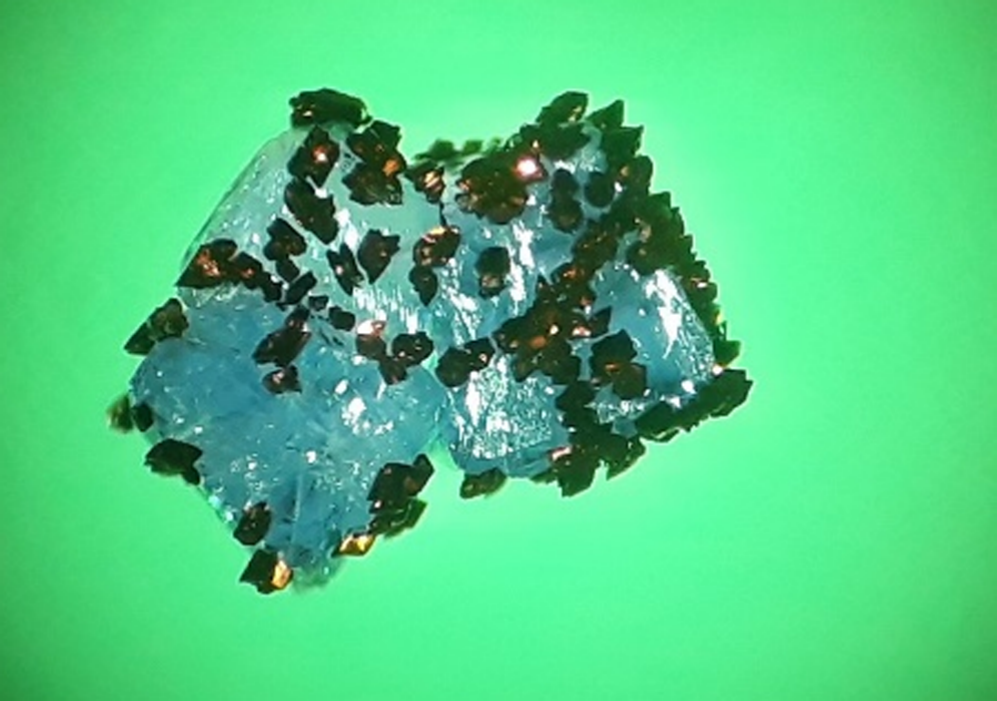
Dark crystals of **1** grown on the surface of blue/purple crystals of [Ni(2*ampy*)_2_(NO_3_)_2_].

Single crystals for the X-ray study were prepared by a horizontal diffusion method as follows:

A few light purple crystals of [Ni(2*ampy*)_2_(NO_3_)_2_] of approximate dimensions 0.7 mm × 0.7 mm × 0.7 mm were added to 3 mL of MeOH in a Petri dish of 30 mm diameter. Then, 3.5 mg of solid LiTCNQ was added about 15 mm away from the purple crystals. A green/blue color developed instantaneously in the solution, and within 10 min, shiny dark crystals of compound **1** appeared on the surface of the purple crystals (Figure 1).

### Synthesis and Crystallization of [*trans-*Zn(2*ampy*)_2_(TCNQ)_2_] (**2**)

The synthesis of **2** was performed by the same procedure as for **1**, using ZnCl_2_ (0.3 mmol; 0.045 g) instead of nickel chloride hexahydrate. Yield: 68%. CHN analysis (exp./calc., in %) for C_36_H_24_N_12_Zn (*M* = 690.04 g/mol): C, 62.24/62.66; H, 3.55/3.51; N, 24.27/24.36. FT-IR (ATR, cm^–1^): 3306m, 3251m, 3052w, 3031w, 2642w, 2173w, 2158vs, 2123w, 1571vs, 1502vs, 1433m, 1382w, 1334vs, 1286w, 1254w, 1212w, 1180vs, 1121s, 1098m, 1016s, 982s, 955w, 930w, 880w, 825vs, 756vs, 718vs, 638s, 541s, 510w, 482vs, 438w.

Single crystals of **2** for the X-ray study were prepared by a similar procedure to that used for the single crystals of **1**, by horizontal diffusion using the complex [Zn(2*ampy*)_2_(NO_3_)_2_] prepared according to Tandon.^34^ Within an hour, dark violet prisms of **2** had formed in the space near the insoluble Zn-containing starting material. These were harvested from the solution and analyzed.

### Synthesis and Crystallization of [Li_2_(TCNQF_4_)(CH_3_CN)_4_]·CH_3_CN (**3**)

**Figure 2:**
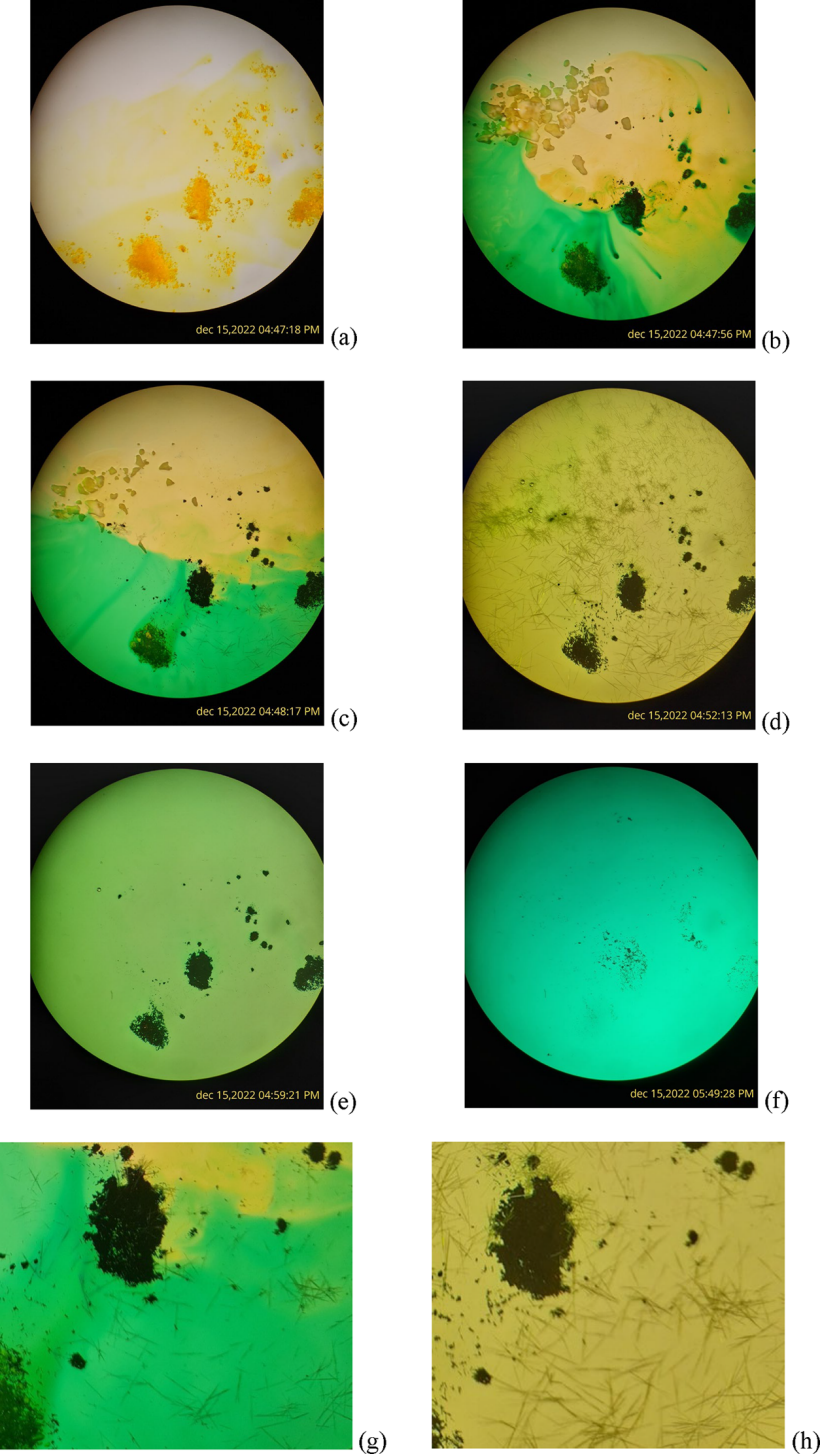
Preparation of single crystals of **3**. In this test, the crystals are visible under the microscope for 1 min after the addition of the reactants, and they begin to disappear about 7 min later. (a–f). Time-stamped progression of crystal growth and disappearance. (g, h) Further magnified views from panels (c, d), respectively.

Single crystals of appropriate size and quality for the X-ray study were prepared by the horizontal diffusion method. In a crystallization dish with a radius of 30 mm were placed 2 mL of acetonitrile, 45 mg (0.36 mmol) of solid LiI, and 2 mg (7.24 × 10^–3^ mmol) of solid TCNQF_4_, with LiI and TCNQF_4_ separated by approximately 15 mm. Within 10 min, transparent needle-like crystals of **3** were formed near the original location of TCNQF_4_. The acetonitrile solution was withdrawn, and FomblinY oil was added immediately in order to cover the crystals and prevent decomposition. The crystals are stable under the oil for a time span of 10–30 min (Figures 2 and S1).

### Instrumental Methods

C, H, and N elemental analyses for **1** and **2** were performed on a PerkinElmer 2400 Series II CHNS/O Analyzer. The infrared spectra for **1** and **2** in the range of 4000–300 cm^–1^ were recorded on a Jasco FT-IR 4600 spectrophotometer using the attenuated total reflectance (ATR) method. The X-ray powder diffraction patterns of bulk samples of **1** and **2** were measured on a RIGAKU D-Max/2500 diffractometer with a rotating anode and an RINT2000 vertical goniometer in the 2θ range 3.0–40° using Cu Kα radiation (λ = 1.5406 Å) and a 2θ step size of 0.02°; the model powder diffraction pattern was calculated using the program *MERCURY*.^35^

### X-ray Structural Analysis of [M(2*amp*y)_2_(TCNQ)_2_] (M = Ni, Zn)

Single-crystal X-ray data for [Ni(2*ampy*)_2_(TCNQ)_2_] (**1**) were collected using the same crystal at 100, 173, and 296 K, and data for [Zn(2*ampy*)_2_(TCNQ)_2_] (**2**) were collected at 100 K on an Oxford Diffraction Xcalibur diffractometer equipped with a Sapphire3 CCD detector and a graphite monochromator utilizing MoKα radiation (λ = 0.71073 Å). The CrysAlis software package^36^ was used for data collection and reduction. Absorption correction was done using ABSPACK (multiscan method).^37^ The structures were solved using SHELXT^38^ and refined against *F*^2^ using the full-matrix least-squares method with the program SHELXL-2018/3^39^ incorporated in the WinGX program package.^40^ Anisotropic displacement parameters were refined for all non-hydrogen atoms. The hydrogen atoms bonded to carbon atoms were included at idealized positions and refined as riders with isotropic displacement parameters assigned as 1.2 times the *U*_eq_ values of their corresponding bonding partners.

The crystal that was used for all three analyses of the Ni(II) complex was found to be an aggregate of two components related by a rotation calculated to be 2.88° about the real-space vector (0.8498, –0.4396, 0.2909) at *T* = 100 K, with a similar relationship holding at the other two temperatures. For the data at 100 K, the refined volume fraction for the second component was 0.369(2). In addition, because of the nature of the twinning, with the accompanying difficulty of separating the intensities of partially overlapped low-angle reflections, and in view of the presence of outliers, after examination of the data and results, it was decided to exclude reflections outside the resolution range of 0.82–5.00 Å (8.15–51.36° 2θ, ShelxL instruction “SHEL 5 0.82”). The low-angle limit excluded 12 reflections in addition to the 11 reflections that were rejected on the grounds of having been, in our judgment, affected by the beamstop. The high-angle cutoff was chosen on the basis of the inspection of quality indicators, including the CC(1/2) statistic.^41^ It was hoped that the refinement of **1** under these conditions would give a result less affected by bias caused by the twinning. Other refinement conditions were also tested, including refinement with no exclusion based on resolution alone; the latter gives *R*_1_ = 0.0697 and w*R*_2_ = 0.1779, so any benefit from fine-tuning the data set in this manner is not reflected in the residuals. For the refinement of **1** at *T* = 296 K, the same resolution limits were used, while for *T* = 173 K, the high-angle limit was not used.

Structure graphics were drawn using the program Diamond.^42^ The crystal data and final parameters of the structure refinements are summarized in Table 1, while selected geometric parameters are given in Table 2. Possible hydrogen bonds are gathered in Table 3.

**Table 1:** Crystal Data and Structure Refinement for [M(2*ampy*)_2_(TCNQ)_2_] [M = Ni (**1**), Zn (**2**)] and [Li_2_(TCNQF_4_)(CH_3_CN)_4_]·CH_3_CN (**3**)^a^

	**1–100**	**1–173**	**1–296**	**2**	**3**
CSD number	2206025	2206024	2206026	2206027	2239060
empirical formula	C36 H24 N12 Ni	C36 H24 N12 Ni	C36 H24 N12 Ni	C36 H24 N12 Zn	C22 H15 N9 F4 Li2
molecular weight	683.38	683.38	683.38	690.04	495.31
crystal system	triclinic	triclinic	triclinic	triclinic	monoclinic
space group	*P*1̅	*P*1̅	*P*1̅	*P*1̅	*P*2_1_/*n*
unit-cell dimensions					
*a* (Å)	9.0246(8)	9.0239(9)	9.0243(7)	9.0137(3)	14.8097(6)
*b* (Å)	9.6591(7)	9.6857(9)	9.7506(7)	9.6468(2)	8.4555(3)
*c* (Å)	10.0936(8)	10.1231(11)	10.2192(9)	10.1775(3)	21.9080(9)
α (°)	89.456(6)	89.085(8)	88.426(7)	90.858(2)	90
β (°)	70.683(7)	70.632(9)	70.606(8)	70.728(3)	109.735(4)
γ (°)	71.792(7)	71.652(9)	71.382(7)	72.056(3)	90
*V* (Å^3^)	784.41(12)	788.41(15)	800.68(12)	788.01(4)	2582.23(18)
*Z*	1	1	1	1	4
*D*_calc_ (Mg/m^3^)	1.477	1.439	1.417	1.454	1.274
*T* (K)	100(2)	173(2)	296(2)	100(2)	103(2)
θ range (°)	4.30–25.67	4.16–28.60	4.13–25.68	2.68–30.91	1.46–25.99
reflections collected	5665	11414	10555	19640	24832
independent reflections	5665	11414	10555	4354	5153
goodness-of-fit on *F*^2^	0.949	0.824	1.035	1.062	1.052
*R* indices (*I* > 2σ_I_)	*R*_1_ = 0.0685	*R*_1_ = 0.0764	*R*_1_ = 0.0696	*R*_1_ = 0.0368	*R*_1_ = 0.0618
	w**R**_2_ = 0.1689	w**R**_2_ = 0.1727	w*R*_2_ = 0.1320	w**R**_2_ = 0.0811	w**R**_2_ = 0.1541
*R* indices (all data)	*R*_1_ = 0.1022	*R*_1_ = 0.1640	*R*_1_ = 0.1490	*R*_1_ = 0.0469	*R*_1_ = 0.0777
	w**R**_2_ = 0.1815	w**R**_2_ = 0.1937	w*R*_2_ = 0.1439	w**R**_2_ = 0.0868	w*R*_2_ = 0.1663
diff. peak/hole (e/Å^3^)	1.619; −0.503	1.287; −0.607	1.016; −0.618	0.375; −0.604	0.790; −0.354

a MoKα radiation [λ(α̅) = 0.71073 Å] was used for all measurements.

**Table 2:** Selected Geometric Parameters for Complexes **1**–**100** and **2** [Å (°)]

	M = Ni, **1**	M = Zn, **2**		M = Ni, **1**	M = Zn, **2**
M-N1	2.102(5)	2.1447(13)	C15-N4	1.151(6)	1.153(2)
M-N2	2.076(5)	2.0797(14)	C17-N5	1.150(6)	1.153(2)
M-N3	2.128(4)	2.2892(14)	C18-N6	1.152(6)	1.154(2)
C14-N3	1.137(5)	1.152(2)	N1-M-N2	80.6(2)	80.09(5)

**Table 3:** Possible Hydrogen Bonds in **1** (**M** = **Ni**) and **2** (**M** = **Zn**) at 100 K [Å (°)]^a^

D–H···A	D–H (**1**/**2**)	H···A (**1**/**2**)	D···A (**1**/**2**)	D–H···A (**1**/**2**)
N2-H2A···N6^ii^	0.91/0.86	2.16/2.18	2.962(6)/2.981(2)	147/155
N2-H2B···N4^iii^	0.91/0.86	2.36/2.35	3.198(6)/3.166(2)	154/157

a Symmetry codes: (ii) *x*, *y* – 1, *z* + 1; (iii) 1 – *x*, 2 – *y*, 1 – *z*.

### X-ray Structural Analysis of [Li_2_(TCNQF_4_)(CH_3_CN)_4_]·CH_3_CN (**3**)

A freshly harvested crystal of [Li_2_(TCNQF_4_)(CH_3_CN)_4_]·CH_3_CN (**3**), covered with a thin layer of perfluorinated oil (FomblinY) and mounted on a MiTeGen micromount, was placed in the nitrogen cold stream of the diffractometer with as little exposure to the ambient atmosphere as was practically possible during the transfer. The cold-stream temperature was maintained at 103 K for the entirety of the diffraction measurements, and the sample showed no signs of decomposition.

Indexing by routine procedures yielded a *C*-centered orthorhombic cell with preliminary dimensions *a* = 14.8027(6), *b* = 41.2186(13), *c* = 8.4409(2) Å, and *V* = 5150.2(3) Å^3^. Data reduction yielded a full data set with *R*_int_ = 0.0517 for the Laue group *mmm*. *R*_int_ is _diffrn_reflns_av-*R*-equivalents as defined in the Core CIF Dictionary (https://www.iucr.org/resources/cif/dictionaries/cif_core viewed on 10 April, 2023). Systematic absences corresponded to the orthorhombic space group *C*222_1_, which has a unique set of absences within the orthorhombic system.

We were unable to derive a recognizable structure solution using orthorhombic symmetry [and not limiting the space group choice to *C*222_1_]; the analysis thereupon proceeded under the hypothesis that the sample was a twin by pseudo-merohedry.^43,44^ In this stage of the analysis, the problem was treated as a case of monoclinic symmetry with pseudo-orthorhombic metrics in the twin. The structure solution and refinement proceeded routinely from that point. For the structure solution, the cell was reduced from the initial *C*-centered orthorhombic *(oC)* setting to primitive monoclinic *(mP),* with the cell parameters given in Table 1. The transformation from *oC* to the *mP* setting, by rows and based on unit-cell axes, is (−1 0 0/0 0 1/0.5 0.5 0), and the twin law for the monoclinic setting, also by rows, is (−1 0 0 / 0 –1 0 / 1 0 1). Both of these matrices can be derived using the program Platon.^45^ On this basis, the original pseudo-orthorhombic *c*-axis of 8.4555(3) Å (final refined value) corresponds to the monoclinic symmetry direction. The first space group choice for the monoclinic setting was *P*2_1_/*n*, based upon “systematically weak” groups of reflections. The twin law for this system obscures systematic absences for putative *n-* or *c-*glide planes since it produces an overlap of absent reflections from one twin individual with nonabsences from the other.

The structure was solved using ShelxD^46^ with space group *P*2_1_/*n* and with the twin information incorporated into the peak list optimization stage of the calculations. The program revealed all 37 non-H atomic sites of the asymmetric unit, and where needed, those sites were assigned their correct element types manually. For the sake of curiosity, we also used ShelxT, which assigned the correct space group, *P*2_1_/*n*, and also revealed all 37 unique atomic sites with their correct elements assigned.

The solution from ShelxD was used to commence the refinement. The first test consisted of isotropic refinement of the 37 non-H atoms, with no twin law applied. This gave *R*_1_ = 0.2345 and *w*R**_2_ = 0.6071, with a significant number of systematic absence violations (356 out of 1132) and with all of the 50 worst-fit reflections having Fo^2^ significantly larger than Fc^2^. This preliminary refinement was repeated with the twin law included, giving *R*_1_ = 0.1166 and *w*R**_2_ = 0.3599 and with 18 of the 50 worst-fit reflections having *F*_o_^2^ < *F*_c_^2^. The population parameter for the second individual refined to a value near one-half. These and other considerations indicate a strong possibility of twinning, as has been expounded by Herbst-Irmer and Sheldrick.^47^

In the final refinement, the non-hydrogen atoms were refined anisotropically, and no restraints or constraints were used for them. H atoms, all of which are in the methyl groups of five acetonitrile fragments, were placed with an idealized geometry about their carrier carbon atoms but with the initial torsion angle about the neighboring C–C bond established by a local difference Fourier calculation. During refinement, the H atoms were treated as riders and also allowed to rotate about the C–C bond but not to tilt.

The refinement converged with the residuals given in Table 1. The final refined value of the population parameter for the second individual was 0.480(2). Selected geometric parameters and possible hydrogen bonds are given in Tables 4 and 5.

**Table 4:** Selected Geometric Parameters for **3** [Å (°)]

C5-N1	1.171(5)	Li1-N1	1.987(7)	N1-Li1-N4	108.3(2)
C6-N2	1.142(5)	Li1-N4	2.001(8)	C2-C1-C3	113.7(2)
C11-N3	1.170(5)	Li1-N5	2.046(6)	C8-C7-C9	113.6(2)
C12-N4	1.150(5)	Li1-N6	2.037(6)		

**Table 5:** Possible Hydrogen Bonds in **3** [Å (°)]^a^

D–H···A	D–H	H···A	D···A	D–H···A
C16-H16A···F1^v^	0.98	2.39	3.245(4)	145
C20-H20C···N3^vi^	0.98	2.68	3.602(5)	157
C22-H22A···F3^vii^	0.98	2.53	3.287(5)	133

a Symmetry codes: (v) *x*, *y* + 1, *z*; (vi) 3/2 – *x*, −1/2 + *y*, 1/2 – *z*; (vii) 1 – *x*, 1 – *y*, −*z*.

### Magnetic Studies

Magnetic properties were investigated using a Quantum Design MPMS-XL5. Measurements of the temperature dependence of the magnetic moment were performed in a magnetic field of 1 kOe in zero-field-cooled (ZFC) and field-cooled (FC) regimes at temperatures from 1.8 to 300 K. The field dependence of magnetization was measured in magnetic fields up to 5 kOe at temperatures of 1.8 and 4.5 K. The diamagnetic contribution of the gelatin capsule, the sample itself (estimated using Pascal’s constants), and the typical value of the temperature-independent paramagnetic susceptibility of Ni^2+^ ions (100 × 10^–6^ emu/mol) for complex **1** were subtracted from the raw data.

## Results and Discussion

### Syntheses, Crystal Formation, and Identification of *trans*-[M(2*ampy*)_2_(TCNQ)_2_] [M = Ni (**1**), Zn (**2**)]

The reaction of LiTCNQ with nickel chloride hexahydrate in the presence of 2*ampy* in a methanolic solution yielded the complex [Ni(2*ampy*)_2_(TCNQ)_2_] (**1**) in a microcrystalline form. With the aim of better understanding the magnetic contribution from the TCNQ ARs, we also synthesized the analogous Zn(II) complex (**2**) (*S* = 0). None of our attempts to recrystallize the microcrystalline solids in order to obtain samples of acceptable size and quality for X-ray diffraction was successful, including using different solvents or diffusion of different combinations of solvents. Moreover, not even the compounds themselves were recovered from these attempts, an indication that once the compounds are in solution, they decompose. This apparently is not unusual in this kind of chemistry, as similar behavior was observed long ago.^25^

A different strategy was designed with the aim of producing the compounds directly in the form of crystals large enough and of sufficient quality for single-crystal X-ray diffraction analysis, thus avoiding recrystallization. However, one of the possible strategies, using a fast reaction, can produce microcrystals unsuitable for single-crystal X-ray diffraction; that is the case for the preparation using MCl_2_, 2*ampy*, and LiTCNQ in MeOH, as described above.

We found that a sufficiently fast reaction that produces single crystals of **1** and **2** that yield successful X-ray diffraction analysis occurs in methanol at room temperature when LiTCNQ is added in close proximity to insoluble crystals of [M(2*ampy*)_2_(NO_3_)_2_] (M = Ni, Zn).^33,34^ The process can be followed under a microscope. Figure 1 shows crystals of **1** grown in about 10 min on the surface of insoluble purple crystals of [Ni(2*ampy*)_2_(NO_3_)_2_].

This is an easy, simple, and, importantly, fast procedure for obtaining crystals, which is often the most challenging step in preparing and characterizing a new product. As it involves a time frame of 10 min as opposed to several hours or even days, we were able to forestall decomposition. This process is not adequate, however, for producing a bulk sample because the relative insolubility of the Ni-containing starting material slows the production of larger quantities of product, once again permitting decomposition or the formation of a mixture of products. Thus, the two procedures, one for synthesizing the bulk product and the other for obtaining single crystals for structure analysis, must both be used. The former is a fast solution-based reaction with soluble reagents, and the latter has soluble LiTCNQ placed in close proximity to crystals of an insoluble precursor. The insolubility of one of the precursors seems to impede the formation of a large number of crystallization nuclei and therefore favors the growth of fewer crystals, as can be seen in Figure 1. To summarize, the method consists of using the reactants in close proximity, with one of them quite insoluble. Obviously, the new compound also has to be insoluble in the solvent used.

The identity of the bulk solids was confirmed by comparing their X-ray powder diffractograms to the calculated patterns based on the single-crystal X-ray structure analyses (see the Supporting Information, Figures S2 and S3). The purity and chemical identity of **1** and **2** were also corroborated by C, N, and H elemental analyses. We note that Ballester *et al.*(23) reported the synthesis and characterization of analogous Ni(II) complexes with TCNQ and tetra N-donor ligands (*cyclam* = 1,4,8,11-tetraazacyclotetradecane, *trien* = 1,4,7,10-tetraazadecane).

The infrared spectra of compounds **1** and **2** (Figures S4 and S5) are dominated by absorption bands located around 2200 cm^–1^ assigned to ν(C≡N) stretching vibrations from TCNQ units. In the case of **1**, two absorption bands at 2178 and 2159 cm^–1^ were observed, while in the case of compound **2**, one additional weaker band was located (2173, 2158, and 2123 cm^–1^). Similar values (2186, 2182, and 2161 cm^–1^) were observed in the IR spectrum of [Ni(*cyclam*)(TCNQ)_2_] with monodentate N-coordinated TCNQ ARs.^23^ Also, in the spectra of both **1** and **2**, other strong absorption bands originating from vibrations of ν(C=C), δ(CH), ω(C(CN)_2_), and ν(C–C) were observed at around 1570, 825, 480, and 1330 cm^–1^; their tentative assignment was done with reference to the literature.^19,48^

### Synthesis and Crystal Formation of [Li_2_TCNQF_4_(CH_3_CN)_4_]·CH_3_CN (**3**)

In order to test the applicability of the simple horizontal diffusion procedure for obtaining crystals of some unstable compounds for which speed can be key, we tested the method with the Li/TCNQF_4_ system. The synthesis of pure bulk “Li_2_TCNQF_4_” in acetonitrile at 50–60 °C has been reported;^32^ however, attempts to obtain crystals were unsuccessful.

Crystals of [Li_2_TCNQF_4_(CH_3_CN)_4_]·CH_3_CN (**3**) can be prepared by the addition of LiI and TCNQF_4_ to acetonitrile under normal laboratory conditions. Figure 2 shows selected photos of the process as viewed under a microscope. The full process is recorded in the Supporting Information (Figure S1). Initially (Figure 2a), the yellow solid TCNQF_4_ is added to acetonitrile in a Petri dish (30 mm diameter); (Figure 2b), after LiI (white cream color, left superior part of the photo of the Petri dish) is added, very quickly (less than 30 s), a dark color appears in the TCNQF_4_ zone. This color is typical of the TCNQF_4_^•–^ radical, which is the first reduction step. In addition, a part of the methanol solution is now green (yellow + dark blue). In less than 1 min, some needles of compound **3** can be observed in the lower right part of the photo in Figure 2c,g. These needles continue appearing, now over the whole Petri dish, for some minutes (Figure 2d,h) and then disappear in approximately 10 min (Figure 2e). Finally, most of the dark blue solid disappears (Figure 2f).

A time span of around 10 min transpires from when the first crystals appear to the disappearance of the last one. We estimate that the “lifespan” of a given crystal, from when it visually appears under the microscope until it disappears, can be from 3 to 6 min. This agrees with the already described instability of “Li_2_TCNQF_4_” in acetonitrile. However, the elimination of the acetonitrile solution (removal by a pipette is sufficient) and the immediate addition of FomblinY oil serves to preserve the crystals long enough to select a single crystal and transfer it to the cold stream of a diffractometer for structure analysis. This simple procedure enables the identification even of species that disappear in a matter of minutes, as in the present case.

Horizontal diffusion techniques are not new,^49,50^ although they do not seem subject to as much use as other methods, except for high-temperature applications.^51^ For the present study, the application of a simple variant of this family of techniques has proved capable of producing direct crystallization with the limited nucleation needed to permit sufficient crystal growth. The method is also easily adaptable in terms of controlling diffusion speed, and importantly, in its simplest form, enabling facile crystal harvesting and mounting.

### Crystal Structures of [M(2*ampy*)_2_(TCNQ)_2_] [M = Ni(**1**), Zn(**2**)]

**Figure 3:**
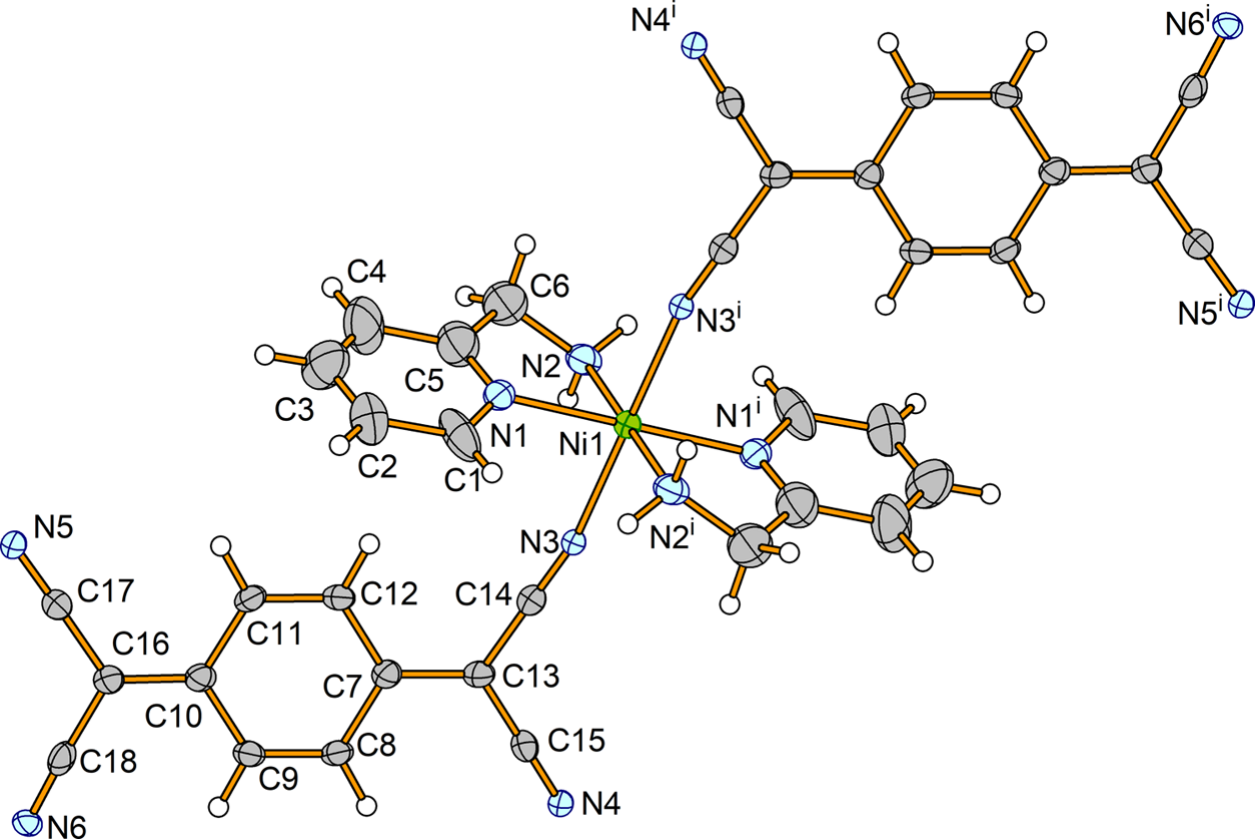
Molecular structure of **1** at 100 K showing the atom numbering scheme. The thermal ellipsoids are drawn at the 50% probability level. Symmetry code: (i) 1 – *x*, 1 – *y*, 1 – *z*.

As the two crystal structures, [Ni(2*ampy*)_2_(TCNQ)_2_] (**1**) (Figure 3 for *T* = 100 K and Supporting Figures S6 and S7 for *T* = 173 and 296 K, respectively) and [Zn(2*ampy*)_2_(TCNQ)_2_] (**2**) (Figure S8), are isostructural, we will describe the crystal structure of **1** and the relevant information for **2** will be given in parentheses. The structure of **1** is formed by molecules of the complex [Ni(2*ampy*)_2_(TCNQ)_2_], which reside on crystallographic centers of symmetry. The central Ni(II) [Zn(II)] atom is coordinated by a distorted octahedron consisting of two chelating 2*ampy* ligands in the equatorial plane and monodentate TCNQ^•–^ ARs in the axial positions. Each axial ligand is coordinated *via* one N atom yielding an {N_6_} donor set. As of this writing, as has been mentioned the CSD^21^ holds three analogous Ni(II) complexes with terminal TCNQ^•–^ ligands *trans-* to each other, namely, [Ni(*bhe*[14]*ane*N_6_)(TCNQ)_2_],^22^ [Ni(*cyclam*)(TCNQ)_2_],^23^ and [Ni((*hd)*-[14]*ane*N_5_)(TCNQ)_2_]·TCNQ·(CH_3_)_2_CO.^24^ In contrast, we do not find any entries in the CSD for an analogous Zn(II) complex with an {N_6_} donor set and two monodentate TCNQ ARs. The nearest similarity to **2** is displayed by the dinuclear complex [Zn_2_(*bpy*)_4_(TCNQ)_2_(TCNQ-TCNQ)] (CUMMOO), in which each Zn(II) atom is coordinated by two chelating 2,2′-bipyridine (*bpy*) ligands and one terminal TCNQ AR with a σ-dimerized (TCNQ)_2_^2–^ dianion bridging the two metals.^52^ We note that in this compound, the nitrogen donor atoms from TCNQ are in *cis*-positions. The Ni–N bond distances in **1** fall in the range 2.076(5)–2.128(4) Å [2.0797(14)–2.2892(14) Å for Zn] (Table 2). These bond distances, as well as other geometric parameters, are in line with those reported for the above-mentioned [Ni(*cyclam*)(TCNQ)_2_] complex at 295 K^23^ and [Zn(TCNQ)(*nicotinamide*)_2_]·DMF at 100 K,^53^ respectively.

It was reported that KTCNQ exists in two polymorphic phases with a first-order phase transition at 122 °C; the low- and high-temperature phases are both monoclinic, but they differ in cell parameters.^54−56^ We explored the possibility of a temperature-dependent phase transition in **1**, which, if present, could be expected to have interesting consequences for the magnetic properties. To this end, we analyzed the structure of **1** at *T* = 100, 173, and 296 K; no qualitative structural change was observed. Selected geometric parameters for **1** at *T* = 173 and 296 K are given in Table S1. We also note that the twinned nature of the crystal did not change with temperature. Selected crystallographic data for these measurements are listed in Table 1, where we observe that the changes are those that are expected with varying temperatures, an example being the unit-cell volume.

**Figure 4:**
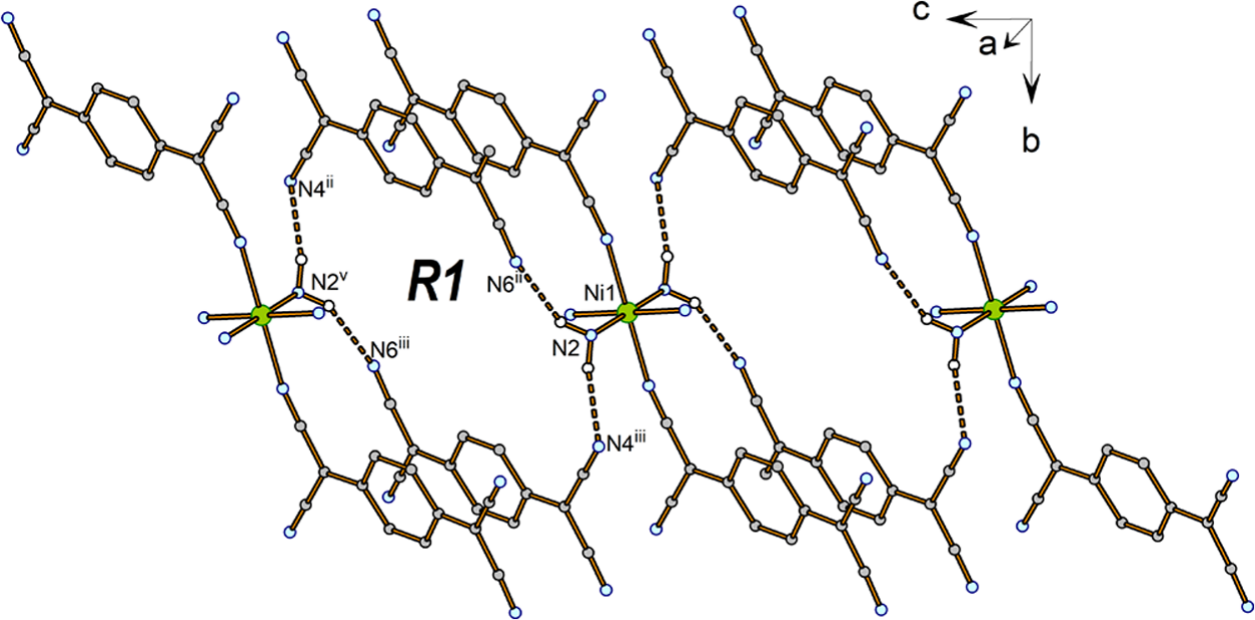
Hydrogen bonding system in **1**. H-bonds are represented as yellow dashed lines. For the sake of clarity, the chelate rings and hydrogen atoms not participating in hydrogen bonds are omitted. Symmetry codes: (ii) *x*, *y* – 1, *z* + 1; (iii) 1 – *x*, 2 – *y*, 1 – *z*; (v) 1 – *x*, 1 – *y*, 2 – *z*.

The extended structures of **1** and **2** are consolidated by N–H···N hydrogen bonds (Figures 4 and S9 and Table 3); these mediate the formation of 2D supramolecular aggregates dominated by hydrogen-bonded ring systems with a graph-set symbol *R*_4_^2^ (26) (Figure 4, ***R1***).

The π-dimerization and consequent spin pairing of the quinoid rings of TCNQ ARs have implications for the magnetic properties of **1** and **2**. The neighboring quinoide rings are coplanar and eclipsed, and the interplanar distances are 3.1236(2) Å f**or 1** and 3.1266(2) Å for **2**. These short distances reflect strong π-dimerization. In the analogous complex [Ni(*cyclam*)(TCNQ)_2_],^23^ a similar distance between planes was observed [3.18(1) Å]. Even shorter distances were found in the compounds [Ni(*trans*-*diene*N_4_)](TCNQ)_2_ (*trans*-*diene*N_4_ = 5,7,7,12,14,14-hexamethyl-1,4,8,11-tetraazacyclotetradeca-4,14-diene) [3.16(1) Å]^57^ and [Ni(*diene*N_4_)](TCNQ)_3_ [3.13(1) Å].^58^

### Crystal Structure of [Li_2_TCNQF_4_(CH_3_CN)_4_]·CH_3_CN (**3**)

**Figure 5:**
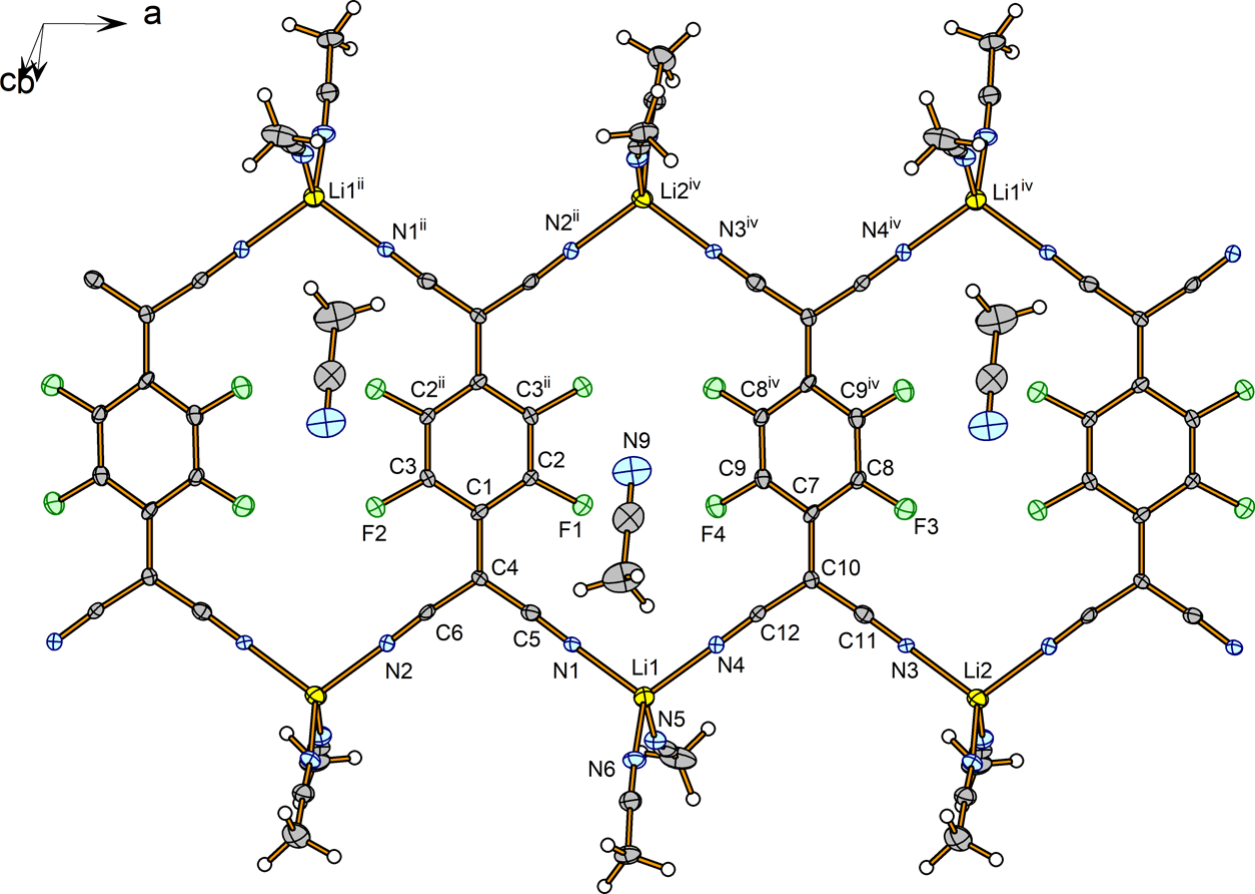
Ribbons of “Li_2_TCNQF_4_” in the crystal structure of [Li_2_TCNQF_4_(CH_3_CN)_4_]·CH_3_CN, (**3**). Symmetry codes: (ii) −*x*, −*y*, −*z*; (iv) 1 – *x*, −*y*, −*z*.

The crystal structure of compound **3** is formed by ribbons of “Li_2_TCNQF_4_” (Figure 5) in which the Li cations form the periphery of the chain and TCNQF_4_^2–^ are the internal part in such a way that the Li cations act as bridges between two TCNQF_4_^2–^ and each TCNQF_4_^2–^ fragment is bonded to four Li cations. The ribbon is thus formed by Li^+^ and TCNQF_4_^2–^ in a 2:1 ratio. The Li cations are also contacted by two terminal CH_3_CN groups, which complete a tetrahedral environment about Li^+^ (Figure 5). There is also one interstitial acetonitrile molecule per TCNQF_4_^2–^. The main distances and angles are collected in Table 4, and the possible hydrogen bonds are in Table 5.

**Figure 6:**
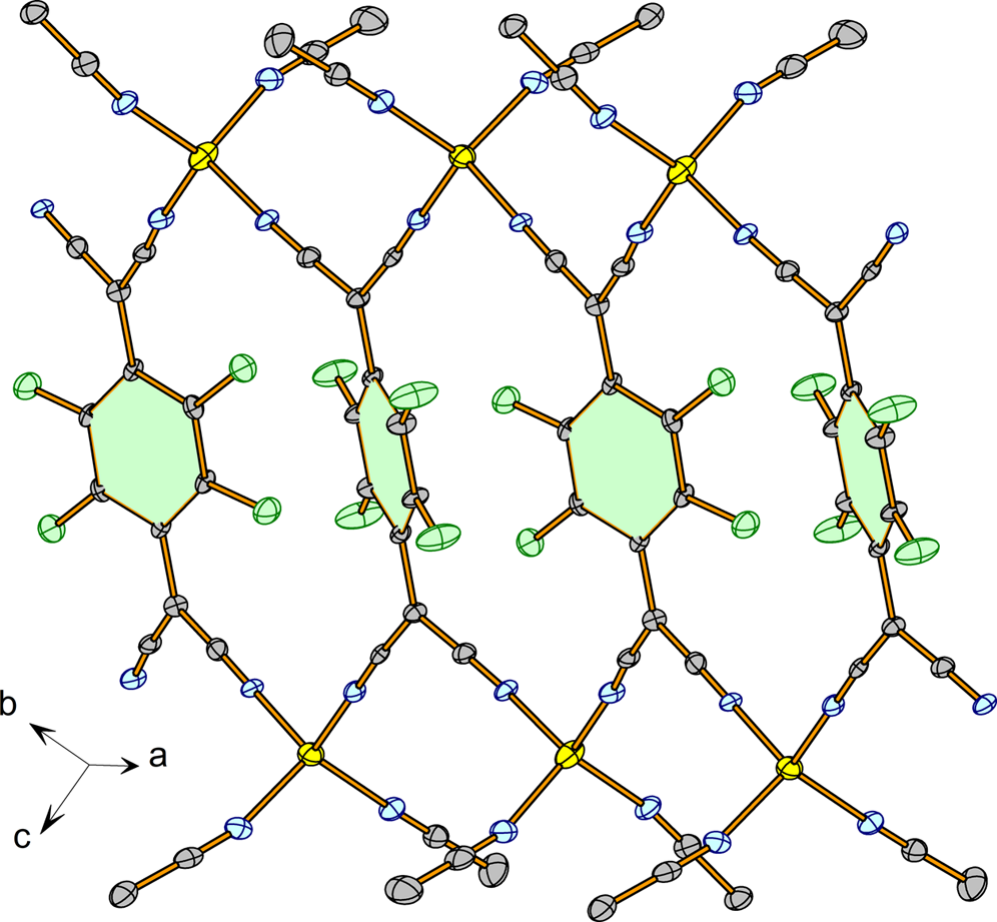
Inclination of the C_6_F_4_ rings in **3**.

**Figure 7:**
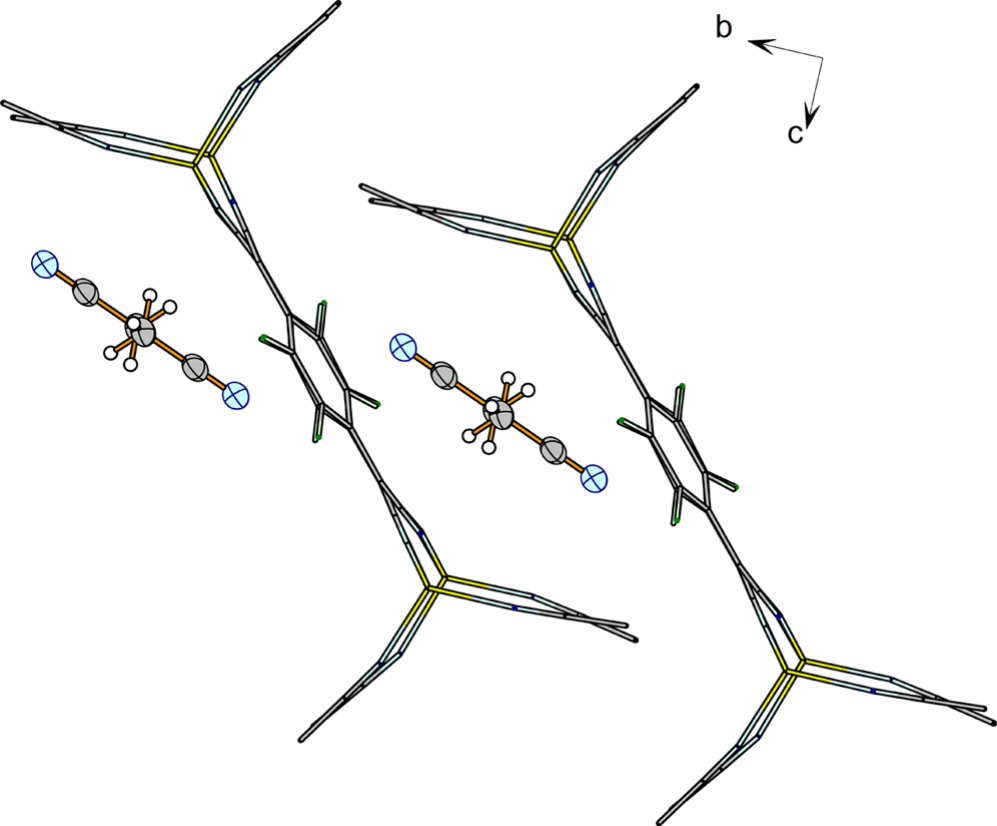
View of the chains in **3**.

Successive C_6_F_4_ fragments along the ribbon are not coplanar, forming a dihedral angle of 35.02(11)°, as can be seen in Figure 6. The chains run parallel to the crystallographic *a-*axis with the interstitial acetonitrile molecules between the chains (Figure 7).

As was mentioned before, there are very few structures with Li and TCNQ or TCNQF_4_, and of those, only in the following cases is TCNQ or TCNQF_4_ bonded to the Li cation. TCNQ^•–^ is bonded to Li[15]crown-5 in the Li^+^([15]crown-5)(TCNQ)_2_^26^ and (15-crown-5)LiTCNQ crystals,^27^ forming discrete aggregations. On the other hand, Li^+^ and TCNQF_4_^2–^ form a 2D aggregate in [Cp_2_Co]Li(TCNQF_4_),^30^ in which Li^+^ is bonded to 4 TCNQF_4_^2–^ groups. Compound **3**, to our knowledge, is the first structurally characterized 1D ribbon formed by Li^+^ and TCNQ^-/2-^ or TCNQF_4_^-/2-^. A related chain can be found in some Cu(I)_2_TCNQ or Cu(I)_2_TCNQF_4_ compounds.^59,60^ In fact, “Cu(I)_2_TCNQF_4_” with acetonitrile has been described;^60^ however, its X-ray single-crystal structure could not be determined, and its structure was related to that of Cu(I)_2_TCNQF_4_(EtCN)_2_, which has CH_3_CH_2_CN instead CH_3_CN. The “Cu(I)_2_TCNQF_4_” chain in the propionitrile compound is similar to the one in compound **3**, although with two interesting differences: (a) the Cu(I) centers have a coordination index of just 3, and (b) the TCNQF_4_ groups in the ribbons are coplanar.

### Magnetic Properties

**Figure 8:**
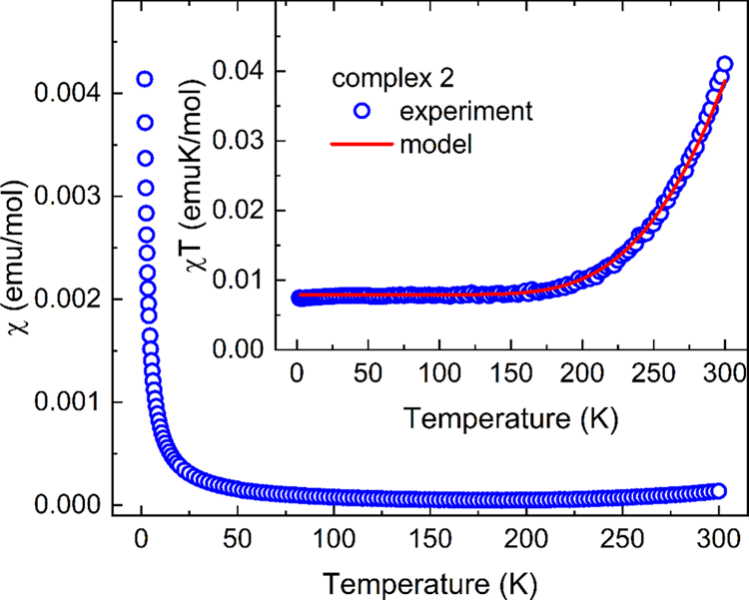
Temperature dependence of susceptibility and χ*T* (inset) of **2** (open symbols) measured in the applied field of 1 kOe, including the model χ = 2(1 – *c)*χ_Curie_ + *c*χ_dimer_ proposed in the text.

**Figure 9:**
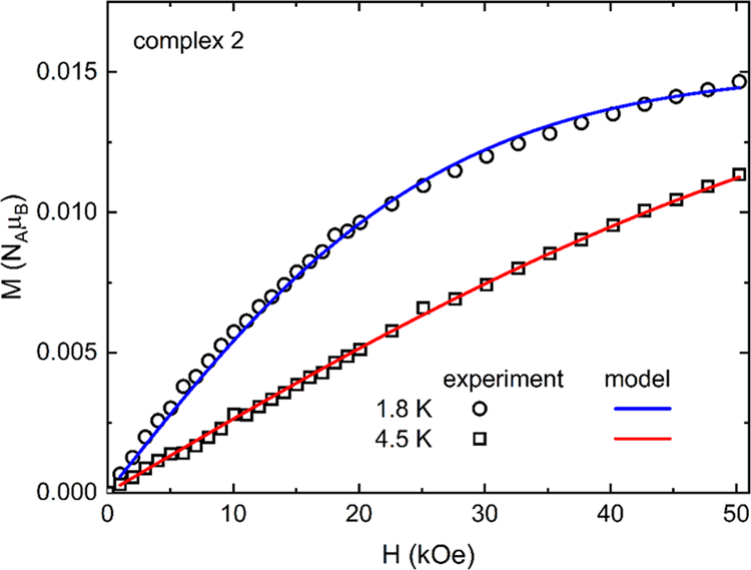
Field dependence of the magnetization of **2** at 1.8 and 4.5 K (open symbols), including the fit of the Brillouin function representing the paramagnetic contribution of nondimerized paramagnetic TCNQ species.

The magnetic properties of **1** and **2** were studied in the temperature range of 1.8–300 K. No difference between ZFC and FC responses was observed, suggesting no magnetic ordering in both complexes. The value of χ*T* = 0.04 emuK/mol of **2** at 300 K (Figure 8, inset), corresponding to the effective magnetic moment μ_eff_ = 0.57μ_B_ (μ_B_ is the Bohr magneton), is evidence of the greatly reduced magnetic moment originating from two TCNQ ARs in a molecule of **2**, each carrying spin 1/2. This reduction is expected due to the presence of strong π-dimerization between TCNQ ARs from neighboring molecules, as shown in Figure S9. The appropriate magnetic model to describe magnetic properties represents an AFM spin dimer with a strong exchange coupling *J*, defined by a Hamiltonian 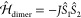
1which allows only a small population of magnetic spin-triplet states to be accessible at ambient temperatures. A small, almost constant value of χ*T* below 200 K can be related to the presence of a minor concentration of nondimerized paramagnetic TCNQ ARs. As a result, the temperature dependence of the susceptibility was described by a model consisting of a paramagnetic contribution described by the Curie law and the Bleaney–Bowers formula^61^ for AFM spin dimers.
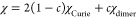
2where parameter *c* represents the reduced concentration of dimers. The best fit to the experimental data was obtained for exchange coupling *J*/*k*_B_ = −1369 K and *c* = 0.995 (only 0.5% of TCNQ AR pairs are noninteracting, contributing to the paramagnetic susceptibility), with the *g*-factor fixed to *g*_TCNQ_ = 2 in all presented calculations. The exchange coupling strength falls between the ones suggested in similar complexes with central Ni(II) ions by Ballester et al.,^23,57^ following the trend in distances between TCNQ ARs from neighboring molecules forming π-dimers. The low-temperature magnetization of **2** shown in Figure 9 can be described by the Brillouin function for the paramagnetic contribution of 0.7% of noninteracting (broken) TCNQ AR pairs (*c* = 0.993).

**Figure 10:**
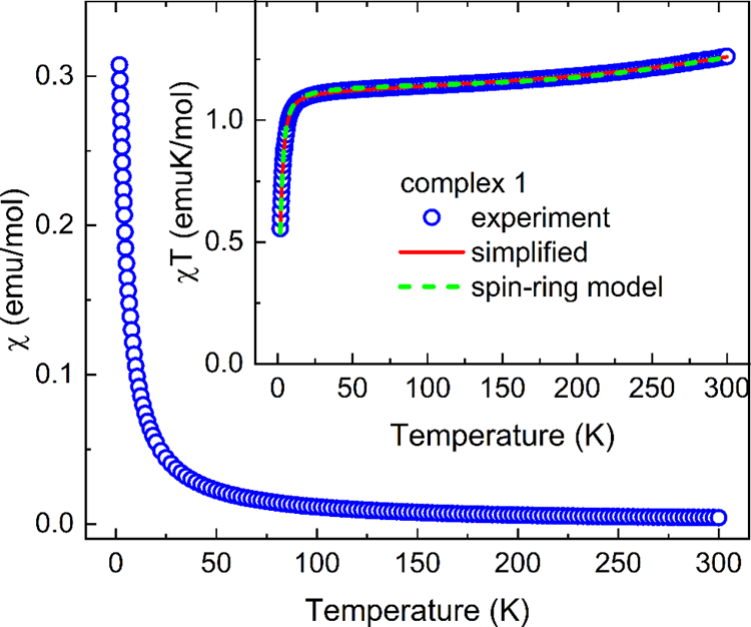
Temperature dependence of the susceptibility and χ*T* (inset) of **1** (open symbols) measured in the applied field of 1 kOe, including the fit of the simplified model χ = χ_Ni_ + χ_dimer_ + χ_0_ (solid line) and a spin-ring model (dashed line) proposed in the text.

Regarding complex **1**, one has to include the contribution of the Ni(II) ion (3d^8^, spin *S* = 1) present in the molecular unit, yielding χ*T* = 1.262 emuK/mol at 300 K (Figure 10, inset), corresponding to the effective magnetic moment μ_eff_ = 3.178 μ_B_. A small increase in χ*T* above 200 K is expected due to the strong π-dimerization between TCNQ ARs from neighboring molecules forming AFM spin dimers. At low temperatures, a drop in χ*T* can be the consequence of zero-field splitting of Ni(II) ions and/or the effective AFM exchange interaction between them. Since at low temperatures, the magnetic moment of Ni(II) ions dominates, a negligible contribution of paramagnetic TCNQ ARs (expected to be similar to **2**) will be omitted in the further analysis. In the first step, we neglected a possible interaction between Ni(II) ions and TCNQ ARs within a molecule, similar to refs (24, 57) but an effective coupling could be transmitted between Ni(II) in neighboring molecules through coordinated TCNQ ARs (π-dimerized TCNQ ARs) and total susceptibility was defined by a simplified model.
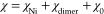
3A possible correction to the estimation of the diamagnetic contribution using Pascal’s constants and temperature-independent paramagnetism is included using χ_0_. The contribution of Ni(II) ions, χ_Ni_, is represented by the formula
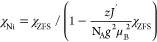
4where *N*_A_ is the Avogadro constant, *g* is the average *g*-factor, *J*′ is an exchange interaction with neighboring *z* ions introduced in the frame of mean-field theory^62^ (in our case, *z* = 2 is assumed), and χ_ZFS_ is the susceptibility of isolated Ni(II) ions described by the effective spin Hamiltonian, taking into account the zero-field splitting (ZFS) with parameters *D* and *E*.
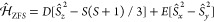
5Since the correct determination of the sign of a small *D* parameter from the analysis of the magnetic response of a polycrystalline sample may be ambiguous, we have used a simple approach to predict its sign and magnitude from the deformation of the coordination octahedron as proposed in ref (63). Using the structural data obtained at 296, 173, and 100 K, one can find that the sign of the *D* parameter changes between 296 and 173 K from positive to negative. Moderate values of *D*/*k*_B_ = 4.9 K at 296 K, *D*/*k*_B_ = −5.4 K at 173 K, and *D*/*k*_B_ = −4.3 K at 100 K were obtained, and a strong rhombicity *E*/*D* is expected. Since the susceptibility is affected by such values of ZFS parameters only at low temperatures, we assumed a single negative *D* parameter as the starting value for our analysis.

**Figure 11:**
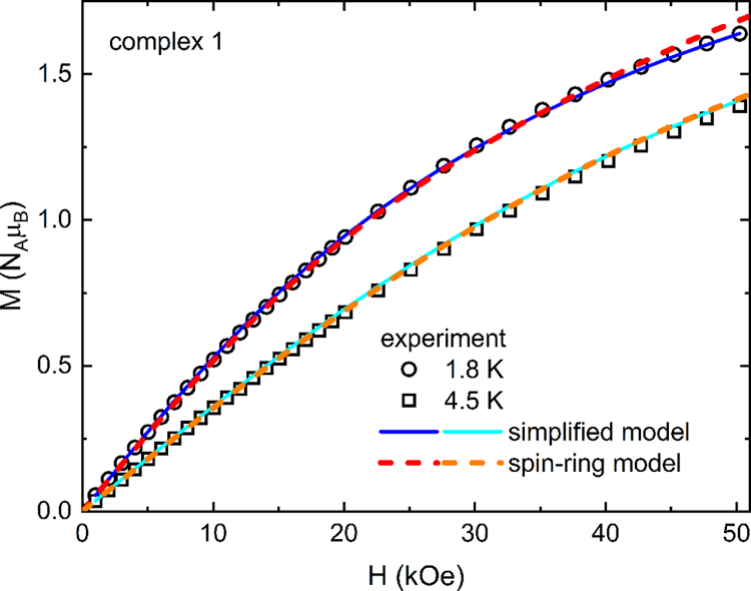
Field dependence of the magnetization of 1 at 1.8 and 4.5 K (open symbols), including the fit of the simplified (solid lines) and spin-ring model (dashed lines) proposed in the text.

The calculation of temperature dependence of the susceptibility χ_ZFS_ and corresponding field dependence of magnetization *M*_ZFS_ based on eq 5 was performed using the EasySpin^64^ toolbox in the MATLAB environment. The average magnetic response was calculated from the distribution of the magnetic-field vector over the orientational grid to account for the polycrystalline characteristics of the sample. The χ*T* was then calculated using eq 3. The contribution of AFM spin dimers in low-temperature magnetization is negligible and was omitted. The calculation of *M*_ZFS_ was performed, also neglecting effective interactions between Ni(II) ions since the inclusion of both the rhombic *E* parameter and effective interaction in the framework of the mean-field theory for the magnetization of a polycrystalline sample is not trivial. The simultaneous fit of χ*T* and magnetization shown in Figures 10 and 11 by solid lines yielded *D*/*k*_B_ = −8.5 K, *E*/*D* = 0.28, average *g*-factor *g*_Ni_ = 2.11, *J*′/*k*_B_ = −0.6 K, *J*/*k*_B_ = −1206 K, and χ_0_ = 3.3 × 10^–4^ emu/mol. A lower value of exchange coupling *J* in **1** in comparison with **2** might suggest some influence of the interaction with neighboring Ni(II) ions on the exchange coupling within the dimerized TCNQ ARs. With the aim of estimating the exchange coupling between Ni(II) ions and TCNQ ARs in the molecule, one would have to define a model Hamiltonian describing an infinite chain of alternating *S* = 1 sites and *S* = 1/2 dimers in the following scheme
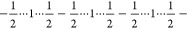
with two exchange couplings, *J* (−) and *J*_1–1/2_ (···). The calculations of magnetic properties for such a model are not available in the literature to our knowledge. We have applied a spin-ring model, taking a fragment of the chain with 2 molecular units  and applying periodic boundary conditions (exchange coupling *J* between the end-chain spins). The Hamiltonian of such a spin ring can be defined in the EasySpin toolbox, including the ZFS parameters for *S* = 1 sites, energy eigenvalues found by the exact diagonalization method, and the susceptibility and magnetization, can be calculated. The best agreement with the experimental data, as shown in Figures 10 and 11 by dashed lines, was then obtained for the set of parameters *D*/*k*_B_ = −6.5 K, *E*/*D* = 0.2, average *g*-factor *g*_Ni_ = 2.12, *J*/*k*_B_ = −1105 K, *J*_1–1/2_/*k*_B_ = 22 K, and χ_0_ = 2 × 10^–4^ emu/mol. A strong ferromagnetic (FM) exchange interaction *J*_1–1/2_ between Ni(II) and ARs was also observed in Ni(TCNQ)_2_^65^ or a Ni(II)-verdazyl radical complex.^66^ It is interesting that the ground state of such a model results in an effective antiparallel orientation of *S* = 1 spins for both possible FM and AFM coupling *J*_1–1/2_, which is in accordance with negative *J*′ obtained from a simplified model of weakly interacting Ni(II) ions and AFM spin dimers.

## Conclusions

A fast and facile variation of well-traveled diffusion methods, consisting of adding the reactants in close proximity in a Petri dish with a solvent in which one of the reactants and the final product are insoluble or only slightly soluble, can produce suitable crystals for X-ray diffraction studies of TCNQ or TCNQF_4_ compounds. This method can be extended to other compounds and can be useful when any of the chemicals is unstable in solution. In addition, intermediate compounds can be identified and isolated when following the reaction under a microscope.

Using this method, the following compounds have been prepared and identified crystallographically: (a) unstable [Li_2_TCNQF_4_(CH_3_CN)_4_]·CH_3_CN (**3**) was prepared and analyzed structurally for the first time showing that Li^+^ and TCNQF_4_^2–^ form a 1D ribbon with two acetonitriles coordinated to each Li^+^ and (b) two new isostructural complexes with TCNQ^•–^ ARs, namely, [Ni(2*ampy*)_2_(TCNQ)_2_] (**1**) and [Zn(2*ampy*)_2_(TCNQ)_2_] (**2**). Both compounds are neutral and formed by a central atom of Ni(II) or Zn(II) coordinated equatorially by two chelating 2*ampy* ligands and in the axial positions by monodentate TCNQ^•–^ ARs.

Measurements of the magnetic susceptibility and magnetization of compounds **1** and **2** were performed. A strong AFM exchange coupling within the pairs of TCNQ^•—^ ARs from neighboring molecules was obtained, *J*/*k*_B_ = −1206 K and *J*/*k*_B_ = −1369 K for **1** and **2**, respectively, from the spin dimer model. The presence of magnetically active anisotropic Ni(II) atoms with *S* = 1 in **1** was included in the model of an infinite chain of alternating *S* = 1 sites and *S* = 1/2 dimers; its simple version, a spin-ring model, was used to estimate the exchange coupling between Ni(II) and TCNQ^•–^ ARs.

## Supporting Information Available

Full process of preparation of single crystals; powder patterns; IR spectra; molecular structures; hydrogen bonding system; and geometric parameters (PDF).

## Abbreviations

*2ampy*: 2-aminomethylpyridine
TCNQ: 7,7,8,8-tetracyanoquinodimethane
IUPAC: 2,2′-(cyclohexa-2,5-diene-1,4-diylidene)dipropanedinitrile
TCNQF_4_: 2,3,5,6-tetrafluoro-7,7,8,8-tetracyanoquinodimethane.
