# Supplementary material for: An Old Crystallization Technique as a Fast, Facile, and Adaptable Method for Obtaining Single Crystals of Unstable “Li2TCNQF4” and New Compounds of TCNQ or TCNQF4: Syntheses, Crystal Structures, and Magnetic Properties
Source: Cryst Growth Des. 2023 May 26;23(6):4357–69. doi: 10.1021/acs.cgd.3c00160 (PMC10251768; doi:10.1021/acs.cgd.3c00160)
Supplement: Supplementary file 1 — cg3c00160_si_001.pdf [file cg3c00160_si_001.pdf]

Supporting Information

An old crystallization technique as a fast, facile and adaptable method for obtaining single crystals of unstable "Li_2TCNQF_4" and new compounds of TCNQ or $TCNQF_4$: Syntheses, crystal structures and magnetic properties.

Slavomíra Šterbinská[a,c], *Mariia Holub*[b]*, *Erik Čižmár*[b], *Juraj Černák*[a], *Lawrence Rocco Falvello*[c]*, *Milagros Tomás*[d].

[a] P. J. Šafárik University in Košice, Faculty of Sciences, Institute of Chemistry, Department of Inorganic Chemistry, Moyzesova 11, 041 54 Košice, Slovakia

[b] P. J. Šafárik University in Košice, Faculty of Sciences, Institute of Physics, Park Angelinum 9, 041 54 Košice, Slovakia

[c] Instituto de Nanociencia y Materiales de Aragón (INMA) and Departamento de Química Inorgánica, CSIC-Universidad de Zaragoza, Zaragoza 50009, Spain

[d] Instituto de Síntesis Quimica y Catálisis Homogénea (ISQCH), Departamento de Química Inorgánica, Pedro Cerbuna 12, University of Zaragoza–CSIC, E-50009 Zaragoza, Spain

Table of contents:

1. Figure S1: The full process of preparation of single crystals of **3**.

2. Figure S2: Powder pattern of **1**.

3. Figure S3: Powder pattern of **2**.

4. Figure S4: IR spectrum of **1**.

5. Figure S5: IR spectrum of **2**.

6. Figure S6. Molecular structure of **1** at 173 K along with atom numbering scheme.

7. Figure S7. Molecular structure of **1** at 296 K along with atom numbering scheme.

8. Figure S8. Molecular structure of **2** at 100 K along with atom numbering scheme.

9. Figure S9. Hydrogen bonding system in **2**.

10. Table S1. Selected geometric parameters for complex **1** at 173 and 296 K [Å, °].

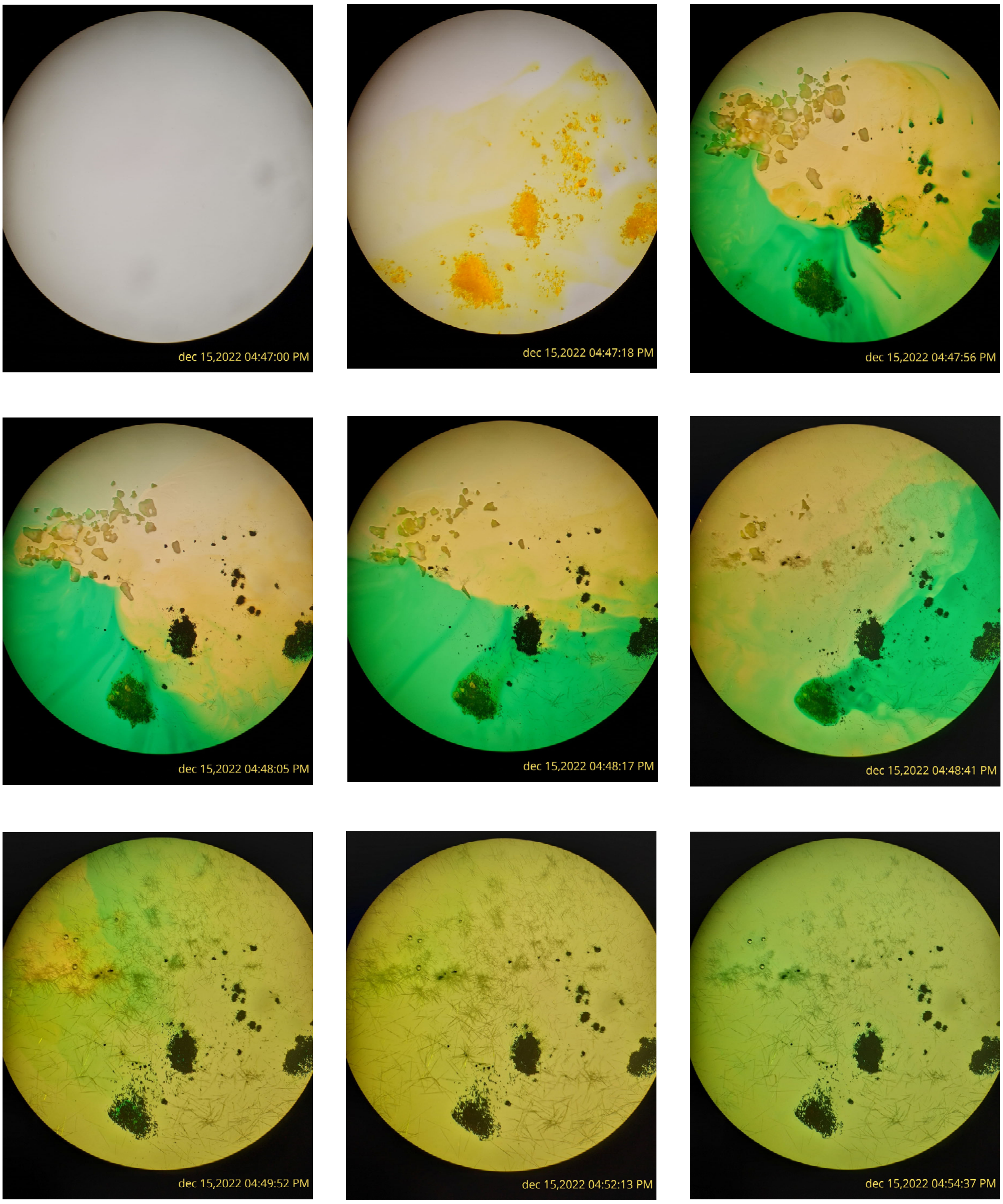
dec 15,2022 04:47:00 PM
dec 15,2022 04:47:18 PM
dec 15,2022 04:47:56 PM
dec 15,2022 04:48:05 PM
dec 15,2022 04:48:17 PM
dec 15,2022 04:48:41 PM
dec 15,2022 04:49:52 PM
dec 15,2022 04:52:13 PM
dec 15,2022 04:54:37 PM

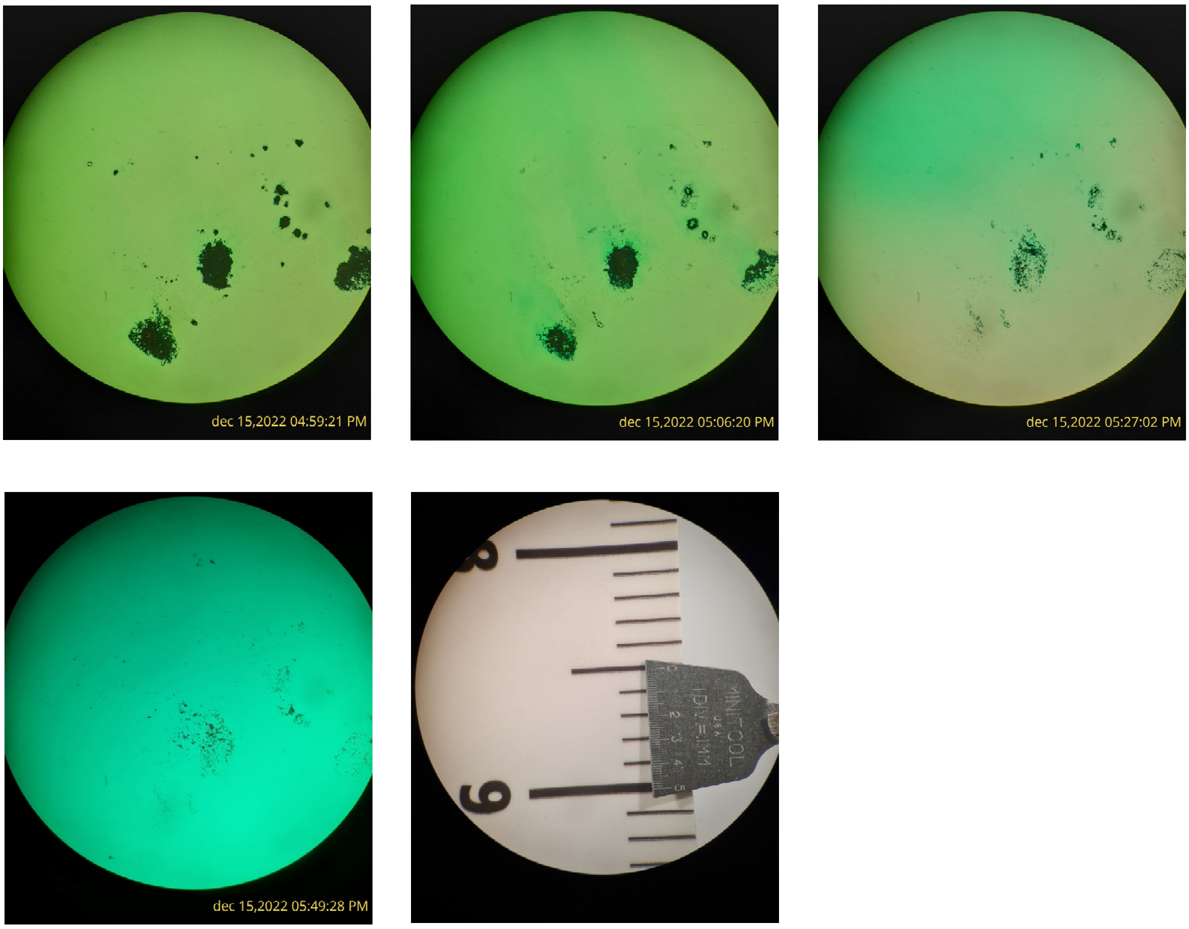

Figure S1. The full process of preparation of single crystals of **3**.

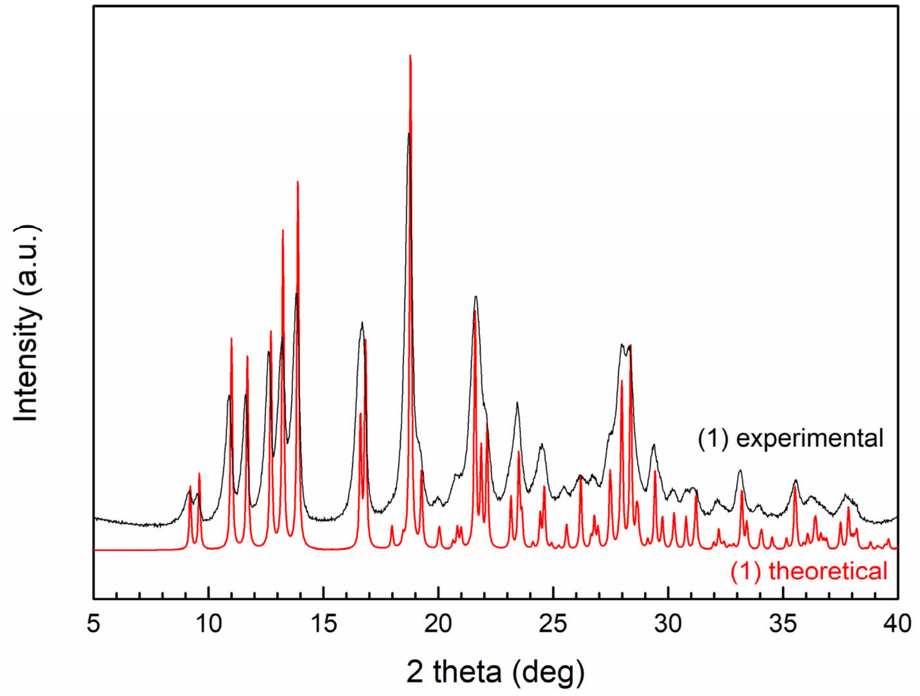

Figure S2. Powder pattern of **1**. The theoretical powder pattern (red) of **1** was based on crystal structure analysis at 296 K and the experimental powder pattern (black) was measured at room temperature.

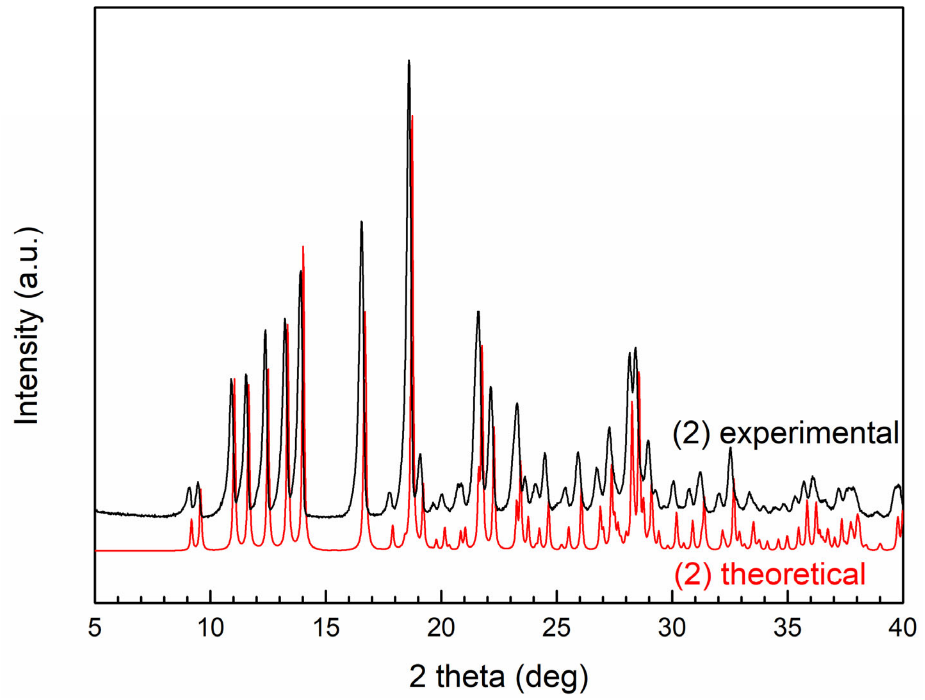

Figure S3. Powder pattern of **2**. The experimental powder pattern (black) was measured at room temperature. The theoretical powder pattern (red) of **2** was based on the atomic parameters from single-crystal structure analysis at 100 K but using the cell parameters determined from a LeBail analysis of the experimental (room temperature) powder pattern.

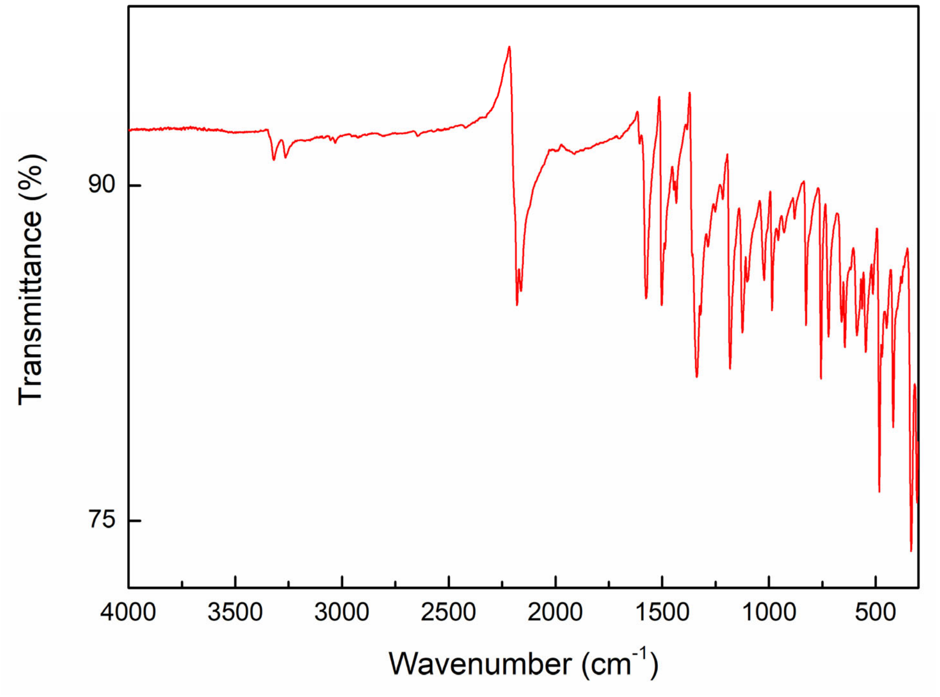

Figure S4. IR spectrum of **1**.

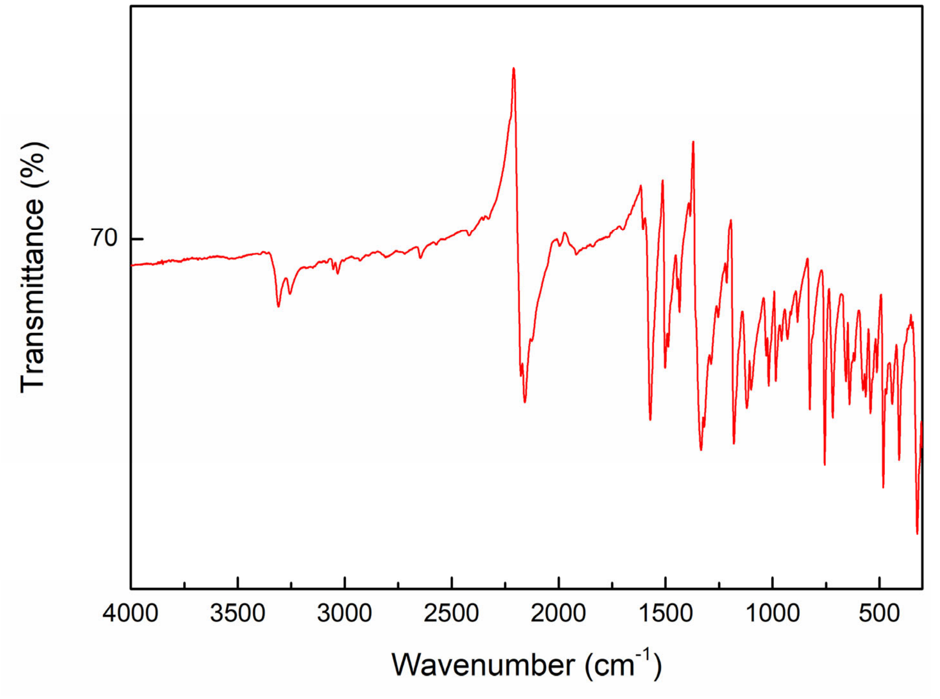

Figure S5. IR spectrum of **2**.

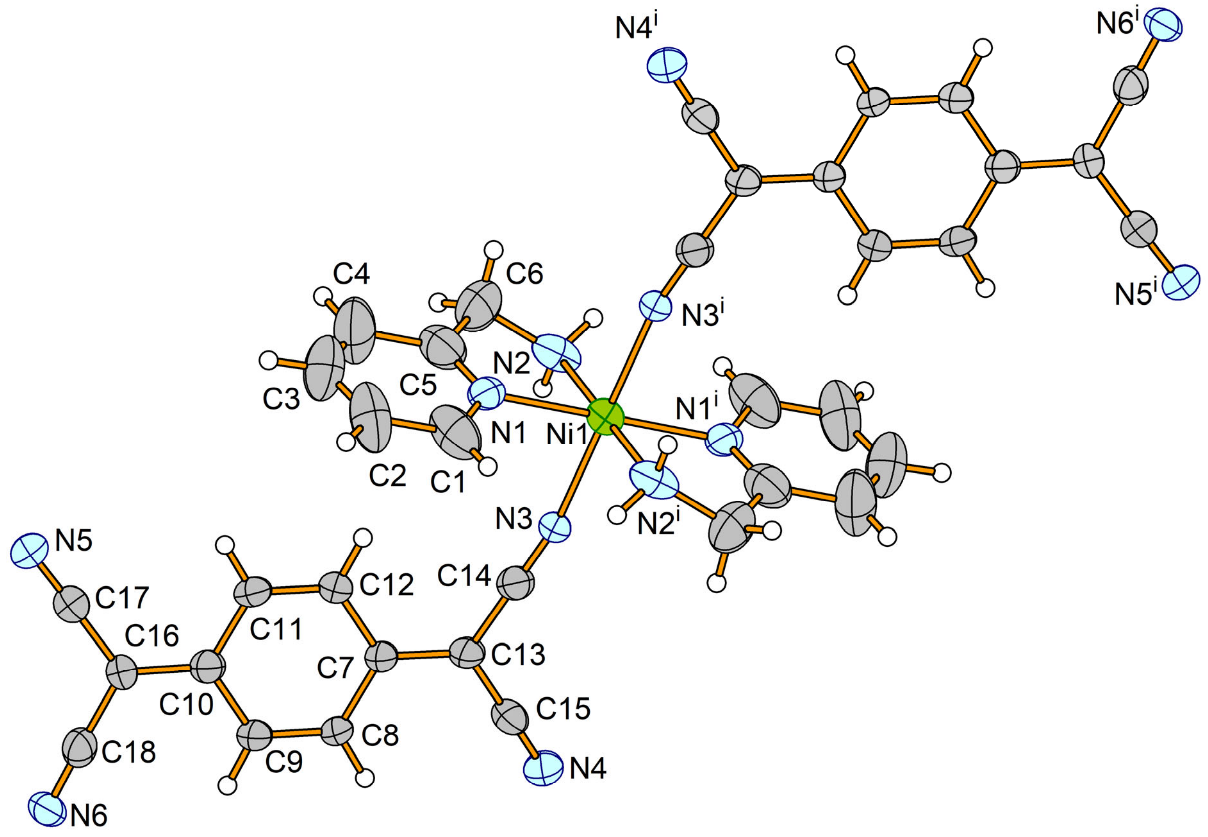

Figure S6. Molecular structure of **1** at 173 K along with atom numbering scheme. The thermal ellipsoids are drawn at 50 % probability level. Symmetry code: i: 1-x, 1-y, 1-z.

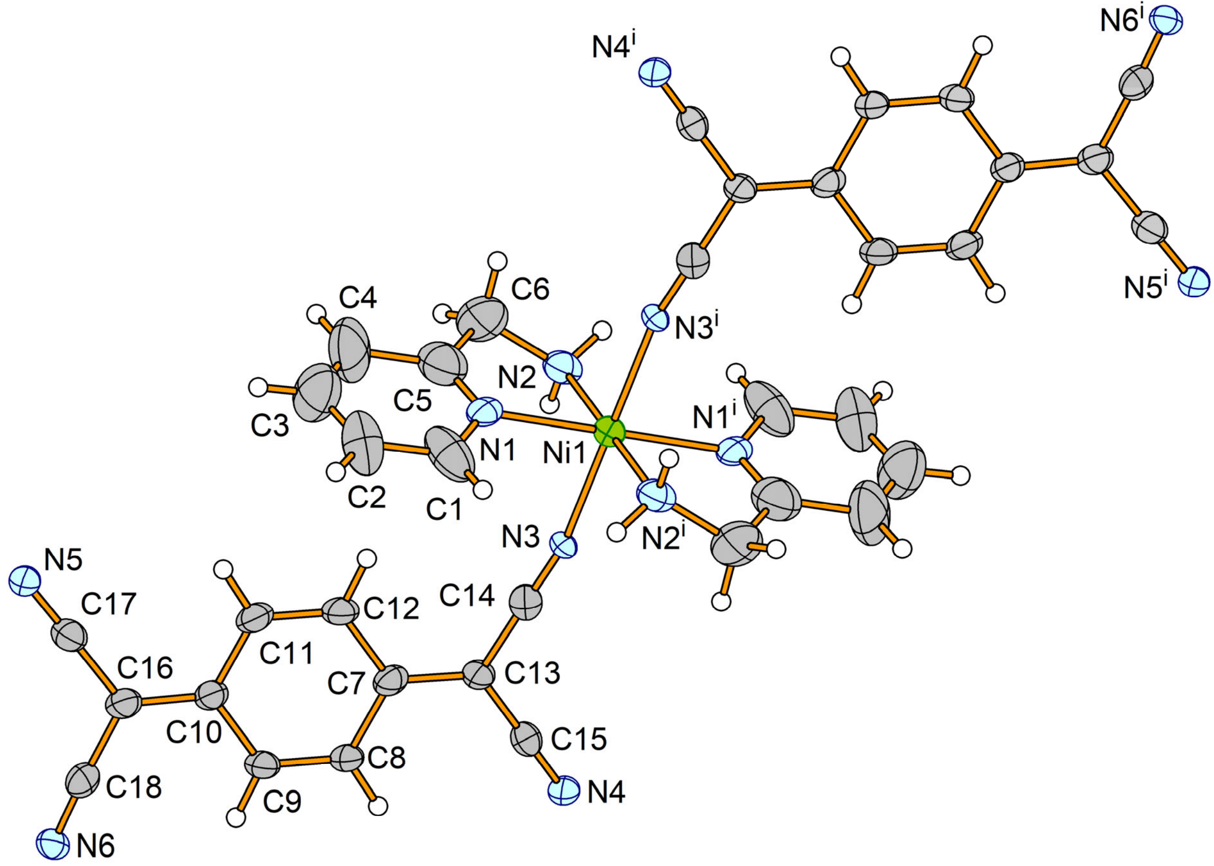

Figure S7. Molecular structure of **1** at 296 K along with atom numbering scheme. The thermal ellipsoids are drawn at 50 % probability level. Symmetry code: i: 1-x, 1-y, 1-z.

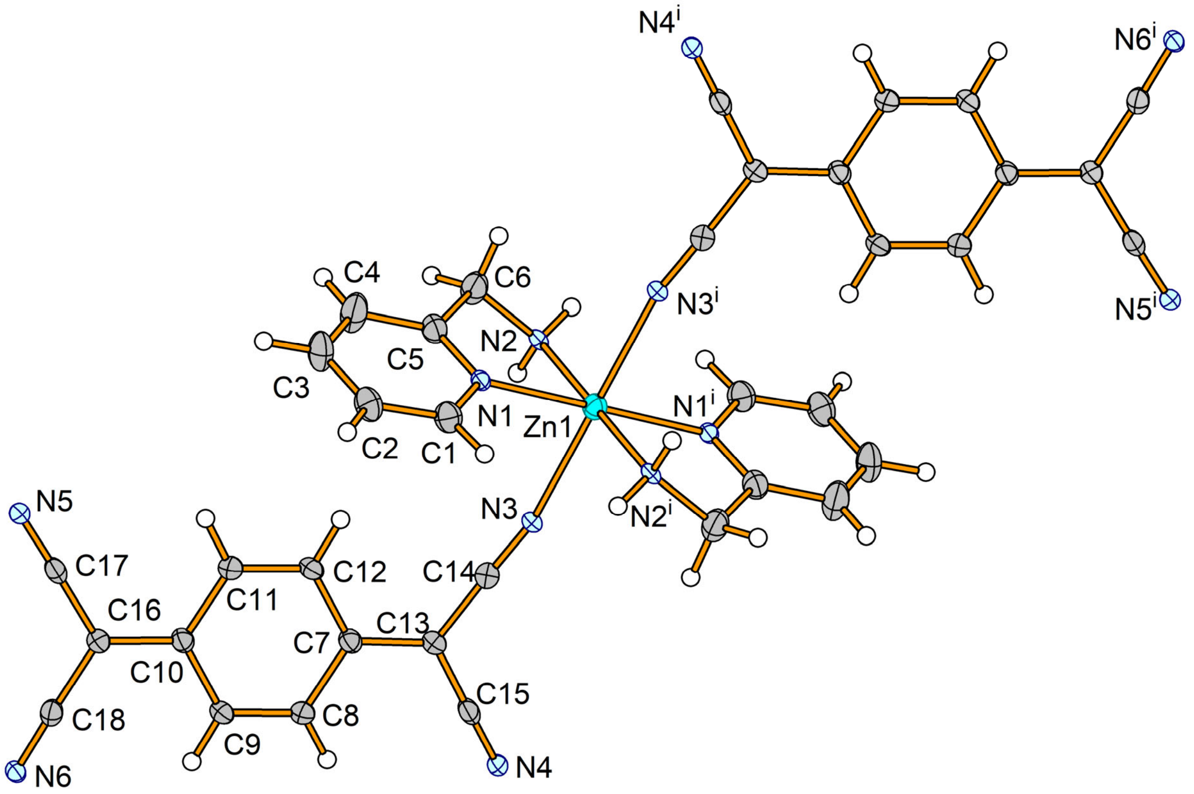

Figure S8. Molecular structure of **2** at 100 K along with atom numbering scheme. The thermal ellipsoids are drawn at the 50 % probability level. Symmetry code: i: 1-x, 1-y, 1-z.

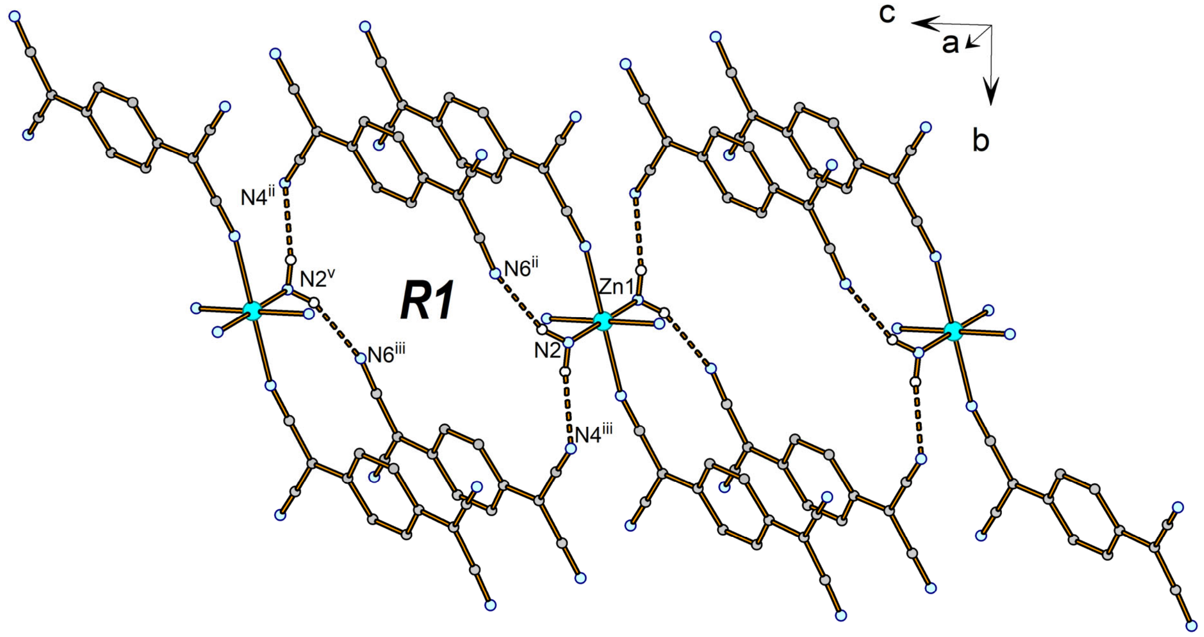

Figure S9. Hydrogen bonding system in **2**. H-bonds are represented as yellow dashed lines. For the sake of clarity the chelate rings and hydrogen atoms not participating in hydrogen bonds are omitted. Symmetry codes: ii: x, y-1, z+1; iii: 1-x, 2-y, 1-z; v: 1-x, 1-y, 2-z.

Table S1. Selected geometric parameters for complexes **1** at 173 and 296 K [Å, °]

	**1**-173	**1**-296		**1**-173	**1**-296
Ni-N1	2.105(6)	2.097(6)	C8-C9	1.344(8)	1.350(7)
Ni-N2	2.064(5)	2.065(4)	C9-C10	1.429(8)	1.420(7)
Ni-N3	2.126(5)	2.129(4)	C10-C11	1.429(7)	1.425(6)
C13-C14	1.421(7)	1.412(7)	C11-C12	1.360(8)	1.355(7)
C13-C15	1.425(9)	1.430(8)	C10-C16	1.393(8)	1.400(7)
C14-N3	1.143(6)	1.151(5)	C16-C17	1.434(8)	1.428(8)
C15-N4	1.157(7)	1.149(7)	C16-C18	1.423(8)	1.427(7)
C7-C12	1.422(7)	1.414(6)	C17-N5	1.142(7)	1.139(6)
C7-C13	1.399(8)	1.404(7)	C18-N6	1.145(6)	1.152(6)
C7-C8	1.430(7)	1.416(6)	N1-Ni-N2	80.7(3)	80.9(2)
